# Additional staining for lymphovascular invasion is associated with increased estimation of lymph node metastasis in patients with T1 colorectal cancer: Systematic review and meta‐analysis

**DOI:** 10.1111/den.14691

**Published:** 2023-10-25

**Authors:** Jun Watanabe, Katsuro Ichimasa, Yuki Kataoka, Atsushi Miki, Hidehiro Someko, Munenori Honda, Makiko Tahara, Takeshi Yamashina, Khay Guan Yeoh, Shigeo Kawai, Kazuhiko Kotani, Naohiro Sata

**Affiliations:** ^1^ Division of Gastroenterological, General and Transplant Surgery, Department of Surgery Jichi Medical University Tochigi Japan; ^2^ Division of Community and Family Medicine Jichi Medical University Tochigi Japan; ^3^ Department of Diagnostic Pathology Tochigi Medical Center Shimotsuga Tochigi Japan; ^4^ Digestive Disease Center Showa University Northern Yokohama Hospital Kanagawa Japan; ^5^ Department of Internal Medicine Kyoto Min‐iren Asukai Hospital Kyoto Japan; ^6^ Section of Clinical Epidemiology, Department of Community Medicine Kyoto University Graduate School of Medicine Kyoto Japan; ^7^ Department of Healthcare Epidemiology Kyoto University Graduate School of Medicine/Public Health Kyoto Japan; ^8^ Scientific Research WorkS Peer Support Group Osaka Japan; ^9^ Division of Gastroenterology and Hepatology Kansai Medical University Medical Center Osaka Japan; ^10^ General Internal Medicine Asahi General Hospital Chiba Japan; ^11^ Department of Gastroenterology and Hepatology, Faculty of Life Sciences Kumamoto University Kumamoto Japan; ^12^ Department of Medicine National University of Singapore Singapore City Singapore; ^13^ Department of Gastroenterology and Hepatology National University Hospital Singapore City Singapore

**Keywords:** colorectal cancer, hematoxylin, lymph node metastasis, meta‐analysis, staining

## Abstract

**Objectives:**

Lymphovascular invasion (LVI) is a critical risk factor for lymph node metastasis (LNM), which requires additional surgery after endoscopic resection of T1 colorectal cancer (CRC). However, the impact of additional staining on estimating LNM is unclear. This systematic review aimed to evaluate the impact of additional staining on determining LNM in T1 CRC.

**Methods:**

We searched five electronic databases. Outcomes were diagnostic odds ratio (DOR), assessed using hierarchical summary receiver operating characteristic curves, and interobserver agreement among pathologists for positive LVI, assessed using Kappa coefficients (*κ*). We performed a subgroup analysis of studies that simultaneously included a multivariable analysis for other risk factors (deep submucosal invasion, poor differentiation, and tumor budding).

**Results:**

Among the 64 studies (18,097 patients) identified, hematoxylin–eosin (HE) and additional staining for LVI had pooled sensitivities of 0.45 (95% confidence interval [CI] 0.32–0.58) and 0.68 (95% CI 0.44–0.86), specificities of 0.88 (95% CI 0.78–0.94) and 0.76 (95% CI 0.62–0.86), and DORs of 6.26 (95% CI 3.73–10.53) and 6.47 (95% CI 3.40–12.32) for determining LNM, respectively. In multivariable analysis, the DOR of additional staining for LNM (DOR 5.95; 95% CI 2.87–12.33) was higher than that of HE staining (DOR 1.89; 95% CI 1.13–3.16) (*P* = 0.01). Pooled *κ* values were 0.37 (95% CI 0.22–0.52) and 0.62 (95% CI 0.04–0.99) for HE and additional staining for LVI, respectively.

**Conclusion:**

Additional staining for LVI may increase the DOR for LNM and interobserver agreement for positive LVI among pathologists.

## INTRODUCTION

Colorectal cancer (CRC) is the third most common cancer worldwide and the second leading cause of cancer‐related death.^1^, ^2^ Two main curative treatments for CRC include endoscopic and surgical resections.^3^ With the advent of advanced endoscopic techniques such as endoscopic submucosal dissection and endoscopic full‐thickness resection, the number of endoscopically treated patients with T1 CRC is increasing.^4^, ^5^ Histopathological evaluation of the risk of lymph node metastasis (LNM) is the key to determining the requirement of complete endoscopic resection.^6^, ^7^ Current treatment guidelines for CRC indicate that T1b (deep submucosal invasion depth [DSI] >1000 μm), lymphovascular invasion (LVI), poorly differentiated adenocarcinoma (PD), indolent cell carcinoma or mucinous carcinoma, and tumor budding (TB) grade 2 or 3 at the site of deepest invasion are risk factors for LNM in CRC.^3^, ^6^, ^8^ Selection of an appropriate treatment, that is, endoscopic versus surgical resection, is critical because surgical resection is associated with the risk of surgery‐related morbidity and mortality, high surgical costs, and reduced patient quality of life, especially in patients with rectal cancer.^3^, ^9^, ^10^

Some previous studies have reported LVI as a strong and reliable predictor of LNM (Table 1).^11^, ^12^, ^13^, ^14^, ^15^, ^16^, ^17^, ^18^ Current guidelines recommend hematoxylin–eosin (HE) staining to diagnose lymphatic and vascular invasion of the tumor.^8^, ^12^ Conversely, a previous study reported that additional staining had a higher diagnostic odds ratio (DOR) for LNM than HE staining (6.0 vs. 2.7).^19^ The use of additional stains, such as D2‐40 for lymphatic invasion (LI) and elastic stains such as Victoria blue (VB) and Elastica van Gieson (EVG) stains for diagnosing vascular invasion (VI), varies among institutions and pathologists.^20^ Some previous studies reported that additional staining had higher interobserver agreements among pathologists than HE staining.^21^, ^22^ However, no large studies or meta‐analyses reported the impact of additional staining for LNM in T1 CRC. The role of additional stains, such as D2‐40 immunostain for LI and elastic stains (VB or EVG stains) for VI, in improving the DOR for LNM and increasing interobserver agreement among pathologists remains unclear.

**Table 1:** Overview of definitions of lymphovascular invasion and details of additional stains

Author, year	Definition of lymphovascular invasion	Additional stains
JSCCR 2010,^11^ 2014,^12^ 2016,^13^ 2019^8^	Lymphatic vessel invasion is defined as the invasion of the tumor cells into the lymphatic vessels. Blood vessel invasion is defined as the invasion of tumor cells into the blood vessels.	When immunostaining is used to examine lymphatic invasion, it should be stated. e.g. Ly1a (D2‐40). When elastic fiber staining is used to examine venous invasion, it should be stated, e.g. V1a (VB) with Victoria blue staining, V1b (EVG) with Elastica van Gieson staining. When a tumor has an elastic plate confirmed at one‐half or more of its circumference, it is considered “V,” and when D2‐40‐positive endothelial cells are confirmed at one‐half or more of its circumference, it is considered “Ly.” Using this method, inconsistencies among individuals judging vascular invasions should be reduced.
World Health Organization^14^	Lymphovascular invasion is defined as the invasion of tumor cells into the blood and/or lymphatic vessels.	NA
The College of American Pathologists^15^	Lymphovascular invasion is classified as not identified, present, or indeterminate.	NA
The Korean Society of Pathologists^16^	Lymphatic or vascular invasion is considered present when tumor cells invade nonmuscle‐walled small vessels or large vessels with a smooth muscle layer and/or an elastic lamina layer.	Immunohistochemical staining with D2‐40 may be performed additionally to identify the endothelial cells because it may be difficult to differentiate the lymphatic vessel from retraction artifacts with HE staining. Special stains for elastic fiber or immunohistochemical stains for CD31, D2‐40, and smooth muscle actin can be used if necessary.

HE, hematoxylin–eosin; JCCRS, Japanese Society for Cancer of the Colon and Rectum; NA, not applicable.

Therefore, this systematic review and meta‐analysis aimed to assess the impact of using additional stains, such as D2‐40 and elastic stains, for LVI on estimating LNM in patients with T1 CRC. In addition, we assessed interobserver agreement among pathologists when employing these immunohistochemical staining methods.

## METHODS

We followed the methodological standard outlines in the Cochrane Handbook for Systematic Review of Diagnostic Test Accuracy and the Preferred Reporting Items for Systematic Review and Meta‐Analysis of Diagnostic Test Accuracy (PRISMA‐DTA) (Appendix S1).^23^, ^24^ We registered the study protocol in the International Prospective Register of Systematic Reviews (PROSPERO, registration number: CRD42022382897) and published this protocol in OSF (https://osf.io/6f5qt/) on December 9, 2022 (Appendices S2,S3).

### Search strategy

We searched the following databases: the Cochrane Central Register of Controlled Trials (CENTRAL), MEDLINE (PubMed), and EMBASE (ProQuest Dialog) from inception until December 10, 2022. Appendix S2 shows the full search strategy. We also searched databases such as the World Health Organization International Clinical Trials Platform Search Portal (ICTRP) and ClinicalTrials.gov for ongoing trials. Our search strategy incorporated keywords associated with CRC, submucosal invasion, and LVI. We included all papers, including published articles, abstracts of conferences, and letters. We did not exclude studies based on the observation period, publication year, language, or country. We reviewed the reference lists of all included studies, international guidelines,^25^, ^26^ and articles citing eligible studies. We clarified additional data from the respective authors.

### Study selection

We included studies that assessed the DORs of additional staining, such as D2‐40 immunostaining for LI and elastic staining (VB or EVG) for VI, and HE staining in patients aged over 18 years with T1 CRC who underwent endoscopic or surgical resection. We excluded case reports, case series, reviews, meta‐analyses, and duplicate cases. The reference standard was the presence of LNM in surgical specimens.

The primary outcomes were the sensitivity, specificity, and DOR of HE and additional staining for LVI to identify LNM in patients with T1 CRC. The secondary outcome was interobserver agreement among pathologists for positive LVI, calculated as Cohen's *κ* of interobserver agreement between two or more pathologists for positive LVI.

### Data collection and risk of bias

Two of six reviewers (J.W. and A.M., H.S., M.H., M.T., or T.Y.) independently screened titles and abstracts and then assessed eligibility based on the full texts and extracted data. We contacted the original authors when relevant data were missing. We resolved disagreements between the two reviewers by discussion, and when this failed, a third reviewer acted as an arbiter (K.I. or Y.K.).

Two of four reviewers (J.W. and A.M., H.S., or M.H.) independently evaluated the risk of bias and applicability using the modified Quality Assessment of Diagnostic Accuracy Studies‐2 (QUADAS‐2) tool.^27^ We revised the signaling questions in the QUADAS‐2 tool according to our present review questions (Appendix S4). We discussed disagreements between the two reviewers, and when this failed, a third reviewer (K.I. or Y.K.) acted as an arbiter. We did not examine publication bias for the DOR of the stains on identifying LNM due to a lack of appropriate statistical methods.^24^

### Data synthesis and statistical analysis

We estimated the sensitivities, specificities, and DORs of HE and additional staining for LNM in T1 CRC in each study using 95% confidence intervals (CIs). In the meta‐analysis, we used bivariate models to calculate the pooled estimates of sensitivity and specificity with 95% CI under the assumption that equal thresholds existed among various stains. The model accounted for within‐study and between‐study variabilities in the accuracy of test performance, including random effects.^28^

We performed a random‐effect meta‐analysis to compare the adjusted ORs for the diagnostic accuracies of additional and HE staining for LVI (an independent risk factor for LNM). This analysis included only studies that incorporated other risk factors (DSI, PD, and TB) in a multivariable analysis. We calculated the *κ* value and standard error using the kappaetc, metan, and metareg modules.^29^, ^30^, ^31^, ^32^, ^33^ We used Review Manager 5.4.1 (RevMan 2020; The Nordic Cochrane Centre, Copenhagen, Denmark) and STATA SE16 software (v. 16.1; StataCorp, College Station, TX, USA) with the midas and metandi packages.^34^, ^35^

### Subgroup and sensitivity analyses

To elucidate the influence of effect modifiers on the results, we performed the following subgroup analyses for the impact of the studied stains for LVI in LNM: (i) EVG vs. VB staining; and (ii) Japan vs. other countries. Subgroup analysis according to additional staining indications (limited cases vs. all cases) was also added. Based on the Cochrane Handbook, we did not use univariate tests for sensitivity and specificity heterogeneity or estimates of the *I* ^2^ statistic because they did not account for heterogeneity explained by phenomena such as positive threshold effects.^24^ However, to assess whether the results of the review were robust to the decisions made during the review process, we performed sensitivity analyses only for the following studies to identify the impact of the stains for LVI in LNM: (i) studies with patients who fully met our inclusion/exclusion criteria (exclusion of studies in which duplicate cases could not be completely ruled out and studies in which additional staining other than D2‐40 immunostaining for LI and elastic staining [VB or EVG] for VI were performed); (ii) those with patients who underwent primary and additional surgical resection; (iii) those with patients who underwent primary surgical resection; and (iv) those that compared additional staining with HE staining in the same patients or institution. We added sensitivity and specificity analyses of CD31 staining for VI.

## RESULTS

### Search results and study characteristics

**Figure 1:**
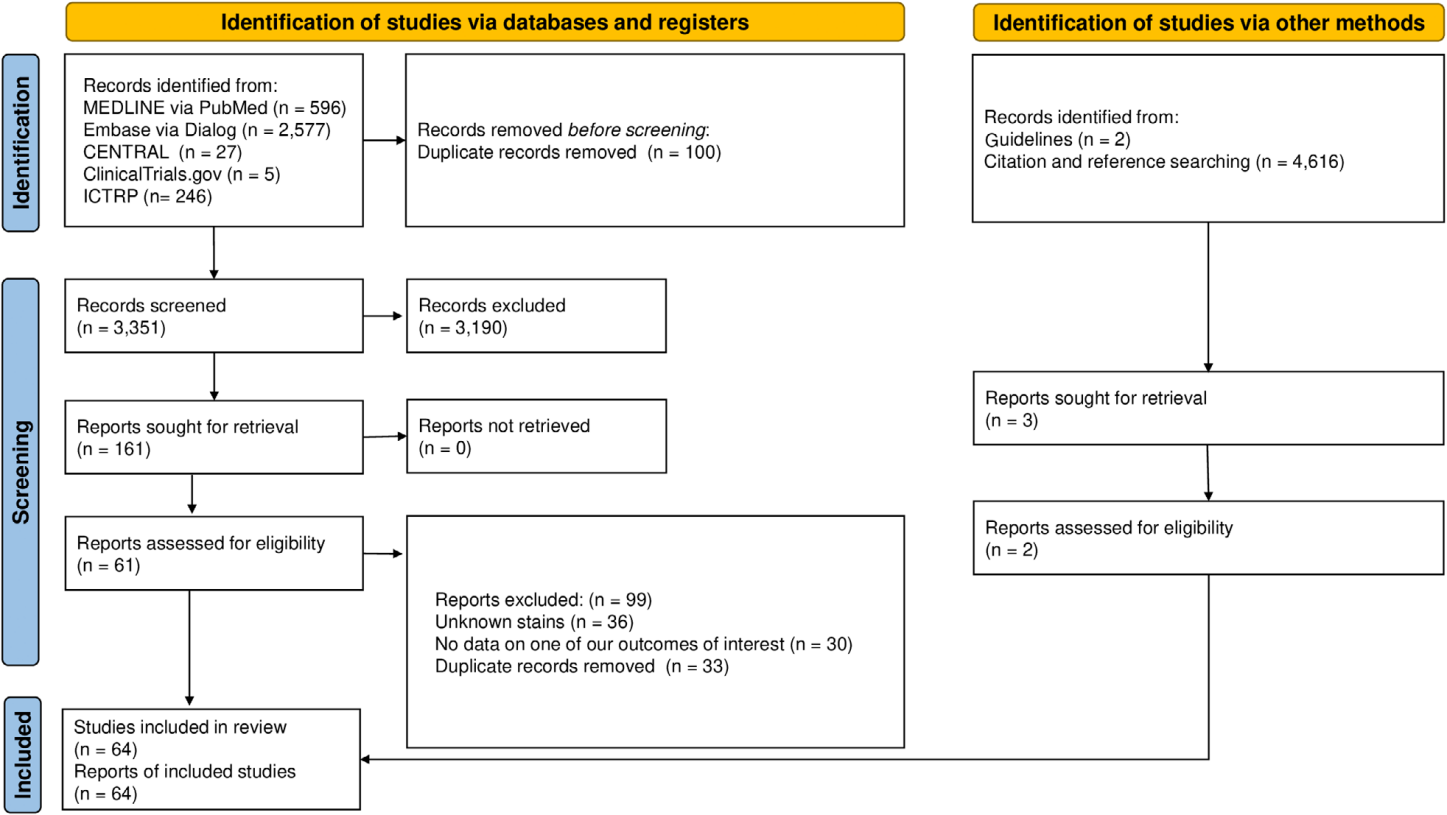
Flowchart of the study selection process.

Figure 1 shows the study selection process. We identified 3451 records from various databases until December 10, 2022. After screening for duplicates, we included 64 studies with 18,097 patients,^17^, ^18^, ^22^, ^36^, ^37^, ^38^, ^39^, ^40^, ^41^, ^42^, ^43^, ^44^, ^45^, ^46^, ^47^, ^48^, ^49^, ^50^, ^51^, ^52^, ^53^, ^54^, ^55^, ^56^, ^57^, ^58^, ^59^, ^60^, ^61^, ^62^, ^63^, ^64^, ^65^, ^66^, ^67^, ^68^, ^69^, ^70^, ^71^, ^72^, ^73^, ^74^, ^75^, ^76^, ^77^, ^78^, ^79^, ^80^, ^81^, ^82^, ^83^, ^84^, ^85^, ^86^, ^87^, ^88^, ^89^, ^90^, ^91^, ^92^, ^93^, ^94^, ^95^, ^96^ of them, 59 studies evaluated the diagnostic accuracies of HE and additional staining for LVI in LNM in patients with T1 CRC, four reported the interobserver agreement among pathologists for positive LVI, and one reported both outcomes, with eight studies comprising 4062 patients in multivariable analysis.

Table S1 presents the study characteristics of the diagnostic accuracies of HE and additional staining for LVI in LNM in patients with T1 CRC: of the total 60 studies, 48 were from Asia with 13,871 patients, 10 from Europe with 3745 patients, one from Russia with 53 patients, and one with a combined Asian and North American cohort of 428 patients. Patients were treated by primary and/or additional surgery in 52 studies and primary and/or additional surgery and endoscopy in eight studies. The indications for surgery in each study were mainly in accordance with Japanese, American, and European guidelines.^3^, ^8^, ^11^, ^12^, ^13^, ^25^, ^26^

Appendix S5 shows the results of the methodological quality assessment of the included studies using the QUADAS‐2 tool. We scored three studies as unclear risks of bias and seven as high applicability concerns for patient selection. We scored 51 studies as unclear risks of bias in the index test, and 11 were scored as high applicability concerns. For reference standard, we scored three studies as high risks of bias and seven as high applicability concerns. For flow and timing, we scored seven studies as high risks of bias and two as unclear risks of bias.

### Diagnostic odds ratio of the stains for LI in LNM

Of the 60 studies, the impact of HE and D2‐40 staining for LI was reported in 24 and 16 studies, respectively; HE and elastic staining for VI was reported in 19 and 21 studies, respectively; and HE and additional staining for LVI was reported in 13 and 8 studies, respectively.

**Figure 2:**
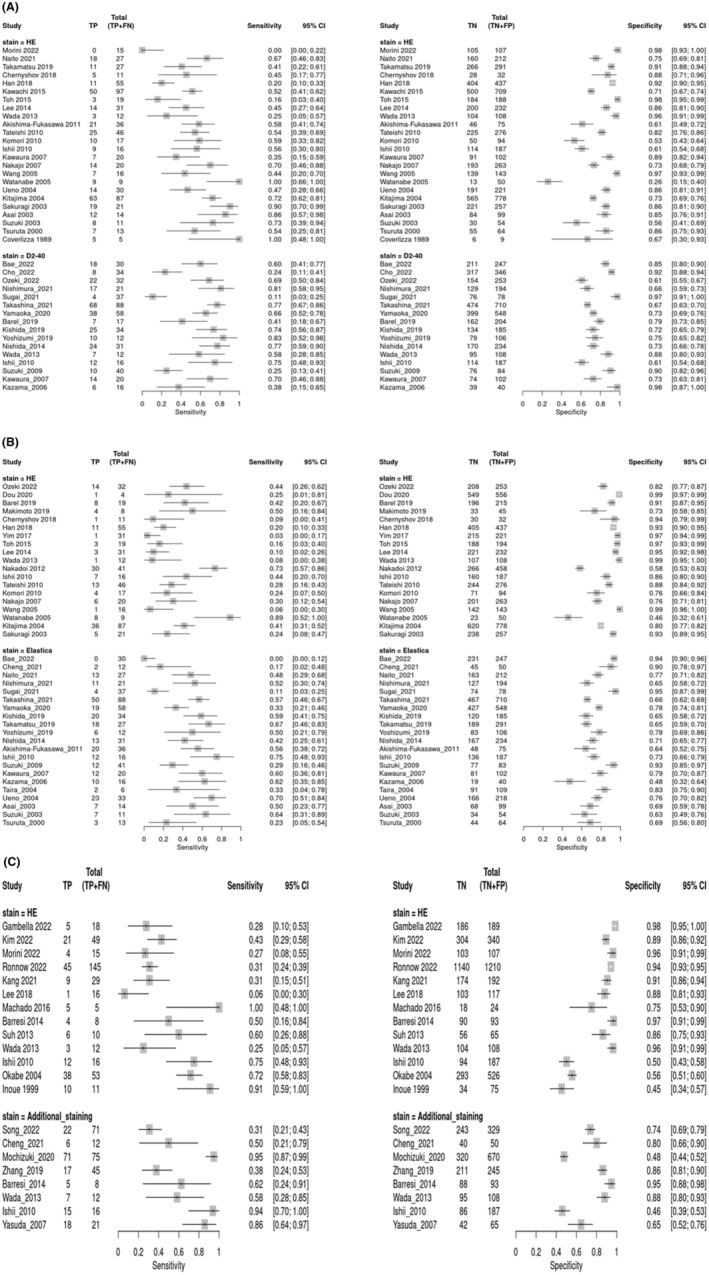
Forest plot with the sensitivities and specificities of (A) hematoxylin–eosin (HE) staining and D2‐40 immunostaining for lymphatic invasion, (B) HE and elastic staining for vascular invasion, and (C) HE and additional staining for lymphovascular invasion on estimating; lymph node metastasis. CI, confidence interval; FN, false negative; FP, false positive; TN, true negative; TP, true positive.

Figure 2A shows pooled sensitivities of 0.53 (95% CI 0.42–0.65) and 0.57 (95% CI 0.44–0.69) (*P* = 0.60), specificities of 0.83 (95% CI 0.75–0.89) and 0.80 (95% CI 0.73–0.86) (*P* = 0.73), and DORs of 5.48 (95% CI 4.17–7.22) and 5.87 (95% CI 4.27–8.06) of HE and D2‐40 staining, respectively, for detecting LI in LNM. Figure 2B reveals pooled sensitivities of 0.25 (95% CI 0.15–0.39) and 0.44 (95% CI 0.34–0.54) (*P* = 0.015), specificities of 0.90 (95% CI 0.83–0.94) and 0.76 (95% CI 0.71–0.81) (*P* = 0.002), and DORs of 3.03 (95% CI 2.32–3.95) and 2.61 (95% CI 2.13–3.20) of HE and elastic staining, respectively, for detecting VI in LNM. Figure 2C indicates pooled sensitivities of 0.45 (95% CI 0.32–0.58) and 0.68 (95% CI 0.44–0.86) (*P* = 0.17), specificities of 0.88 (95% CI 0.78–0.94) and 0.76 (95% CI 0.62–0.86) (*P* = 0.14), and DORs of 6.26 (95% CI 3.73–10.53) and 6.47 (95% CI 3.40–12.32) of HE and additional staining, respectively, in identifying LVI in LNM.

**Figure 3:**
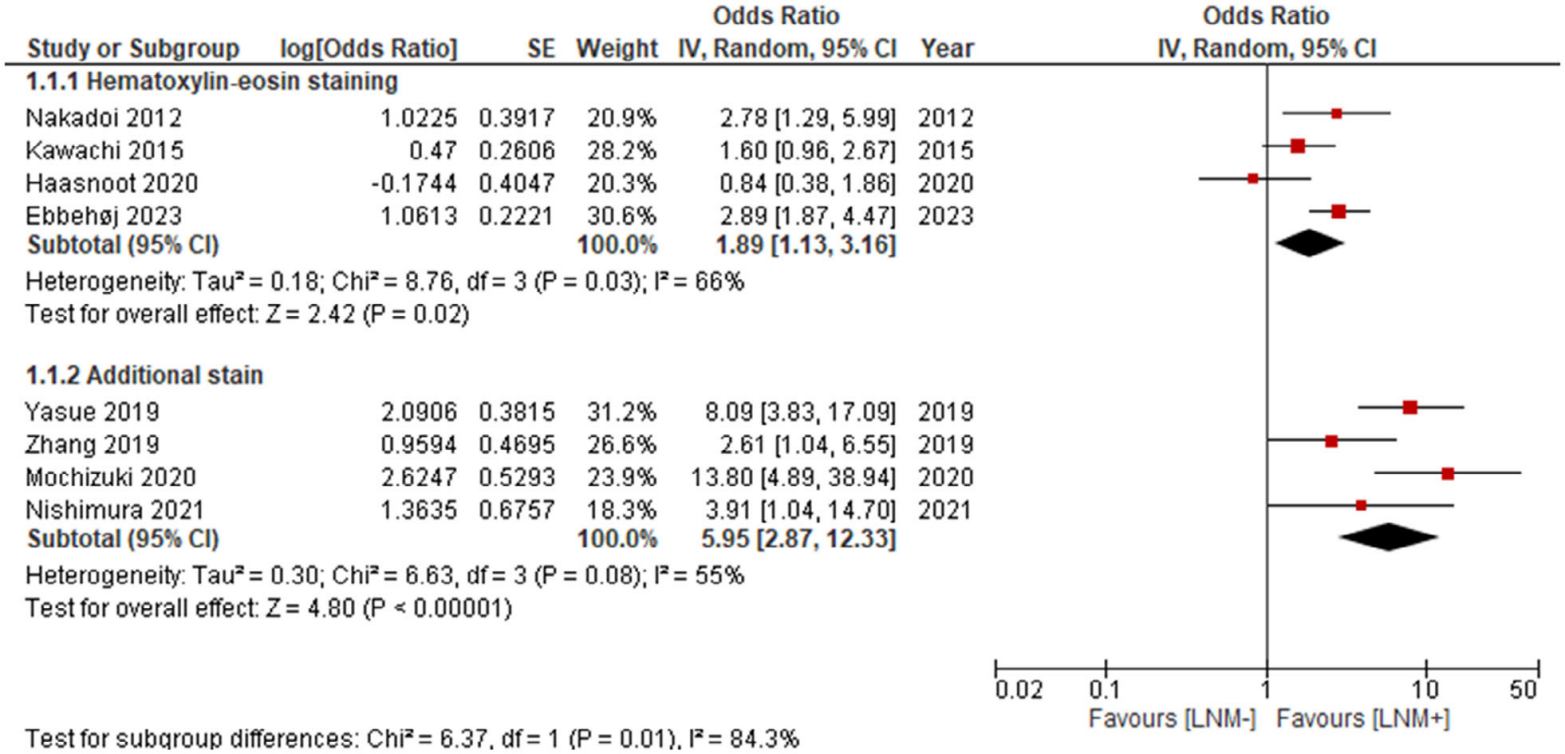
Forest plot with the odds ratios of hematoxylin–eosin and additional staining in studies that simultaneously included other risk factors (deep submucosal invasion, poor differentiation, and tumor budding) in multivariable analysis. CI, confidence interval; IV, inverse variance; LNM, lymph node metastasis; SE, standard error.

In multivariable analysis, the DOR for LVI in LNM differed between the additional (DOR 5.95; 95% CI 2.87–12.33) and HE (DOR 1.89; 95% CI 1.13–3.16) (*P* = 0.01) staining (Fig. 3).

### Additional analysis

Figure S1 displays the subgroup analysis of different staining methods for VI: pooled sensitivities of 0.36 (95% CI 0.22–0.53) and 0.54 (95% CI 0.44–0.64) (*P* = 0.14), specificities of 0.80 (95% CI 0.73–0.86) and 0.70 (95% CI 0.62–0.78) (*P* = 0.11), and DORs of 2.36 (95% CI 1.70–3.26) and 2.77 (95% CI 1.89–4.06) of EVG and VB staining, respectively, for VI in LNM.

Figure S2 shows the country‐based subgroup analysis: HE staining for LVI in LNM had pooled sensitivities of 0.57 (95% CI 0.31–0.80) and 0.38 (95% CI 0.25–0.54) (*P* = 0.15), specificities of 0.78 (95% CI 0.47–0.94) and 0.92 (95% CI 0.87–0.95) (*P* = 0.10), and DORs of 4.62 (95% CI 2.58–8.30) and 6.54 (95% CI 4.47–9.57) in Japan and other countries, respectively. Additional staining for LVI in LNM showed pooled sensitivities of 0.88 (95% CI 0.69–0.96) and 0.41 (95% CI 0.29–0.55) (*P* = 0.02), specificities of 0.64 (95% CI 0.43–0.80) and 0.85 (95% CI 0.75–0.92) (*P* = 0.47), and DORs of 10.27 (95% CI 4.68–22.55) and 3.00 (95% CI 1.15–7.80) in Japan and other countries, respectively.

Figure S3 presents the subgroup analysis of additional staining indications: D2‐40 staining for LI in LNM had pooled sensitivities of 0.59 (95% CI 0.44–0.72) and 0.54 (95% CI 0.28–0.77) (*P* = 0.73), specificities of 0.81 (95% CI 0.73–0.87) and 0.78 (95% CI 0.59–0.90) (*P* = 0.72), and DORs of 6.22 (95% CI 4.67–8.28) and 4.09 (95% CI 2.60–6.42) in all and limited cases, respectively. Elastic staining for VI in LNM showed pooled sensitivities of 0.41 (95% CI 0.30–0.55) and 0.55 (95% CI 0.37–0.71) (*P* = 0.36), specificities of 0.77 (95% CI 0.69–0.83) and 0.74 (95% CI 0.70–0.78) (*P* = 0.53), and DORs of 2.34 (95% CI 1.70–3.22) and 3.06 (95% CI 1.75–5.37) in all and limited cases, respectively. Additional staining for LVI in LNM showed pooled sensitivities of 0.80 (95% CI 0.58–0.92) and 0.34 (95% CI 0.26–0.43) (*P* = 0.03), specificities of 0.75 (95% CI 0.56–0.87) and 0.79 (95% CI 0.76–0.82) (*P* = 0.66), and DORs of 11.66 (95% CI 6.45–21.07) and 2.03 (95% CI 1.10–3.74) in all and limited cases, respectively. Results of the sensitivity analyses were consistent with the main results. Table S2 shows detailed results of the sensitivity analyses.

### Interobserver agreement among pathologists for LVI

Five studies reported interobserver agreement among pathologists for positive LVI. For LI, the overall estimate of Cohen's *κ* was 0.30 (95% CI 0.16–0.44) with HE staining and 0.50 (95% CI 0.37–0.63) with D2‐40 staining (beta‐coefficient 0.20; 95% CI −0.22 to 0.61; *P* = 0.175). For VI, the overall estimate of Cohen's *κ* was 0.30 (95% CI 0.16–0.44) with HE staining and 0.50 (95% CI 0.37–0.63) with D2‐40 staining (beta‐coefficient 0.37; 95% CI 0.0057–0.73; *P* = 0.049). For LVI, the overall estimate of Cohen's *κ* was 0.37 (95% CI 0.22–0.52) with HE staining and 0.62 (95% CI 0.04–0.99) with additional staining (beta‐coefficient 0.21; 95% CI −0.47 to −0.90; *P* = 0.433).

## DISCUSSION

This systematic review and meta‐analysis of 64 studies with 18,097 patients investigated the impact of additional stains, such as D2‐40 and elastic stains, on estimating LNM in patients with T1 CRC. We found that the DORs of these additional staining methods may be higher than that of the HE staining method in multivariable analysis after adjusting for major risk factors (DSI, PD, and TB). We also demonstrated that additional staining may increase interobserver agreement among pathologists for positive LVI. These findings indicate that the addition of the D2‐40 immunostain for LI and elastic stains for VI, such as VB or EVG, may enhance the DOR of LVI for LNM compared with HE stains alone.

In our meta‐analysis of multivariable studies, the DOR of additional staining for LNM was higher than that of HE stains. In previous systematic reviews, LVI was an independent risk factor for LNM in T1 CRC. However, most of these studies did not provide an exact definition of LVI, and histopathological methods varied among these studies,^17^, ^97^, ^98^ leading to a lack of standardization and bias.^17^, ^97^, ^98^ In the present study, the histopathological methods were also evaluated. We found that, in some studies, the combination of LI and VI into one factor may result in a lack of information and a lower risk of LNM.^17^, ^97^ Therefore, it is important to evaluate LVI separately from LI and VI. In the present study, LI and VI were also examined separately, suggesting that additional staining, especially for VI, may affect the DOR of LNM. The reason for the difference in improved DOR of additional staining methods for LI and VI is unclear, but since it is often difficult to distinguish small lymph vessels and venous vessels, additional staining for VI may have particularly improved the DOR. The additional staining enables the distinction between LI and VI, which is difficult with HE staining alone.

With the expanded indication for endoscopic resection as the first‐line treatment in T1 CRC, we recommend additional staining to enhance the DOR for LNM, thereby optimizing patient management. Guidelines recommended considering risk factors, such as DSI, LVI, PD, and TB, when determining the need for a secondary surgical resection after endoscopic resection.^8^, ^14^ In a previous meta‐analysis, DSI was a poor predictor of LNM, while LVI, PD, and TB had high diagnostic accuracies for LNM.^98^ Considering that in the present meta‐analysis, the DORs for LVI in LNM differ between additional (DOR 5.95; 95% CI 2.87–12.33) and HE (DOR 1.89; 95% CI 1.13–3.16) staining, it is possible that additional staining may be more useful than HE staining alone. The identification of LVI using additional staining as one of the most reliable predictors of LNM (i.e. by including it in the training data) will ultimately contribute to improving the DOR of LNM prediction models in combination with other factors such as artificial intelligence and nomograms.^99^

The Japanese Society of Colorectal Cancer and the Korean Society of Pathology recommend additional staining methods, but the World Health Organization and the American Society of Pathology do not.^8^, ^11^, ^12^, ^14^, ^15^, ^16^ Differences in the recommendation of additional staining methods by the pathological societies in each country may have affected the difference in the DOR of additional staining between Japan and other countries in the subgroup analysis of this study. In a study showing pathological diagnostic criteria developed using the Delphi method, the histological findings associated with LVI using HE stains were inconsistent. However, the observations of elastic‐stained internal elastic membrane and D2‐40‐stained endothelial cells covering more than half of the circumference surrounding the tumor cluster were highly consistent.^21^ The present study revealed a higher interobserver agreement among pathologists with additional staining for LI, VI, and LVI than with HE staining alone in patients with T1 CRC. Although uniform criteria strongly influence the consistency of the diagnosis of LVI, pathologists from different countries may interpret the diagnostic criteria for LVI in CRC differently.^22^

The findings of this study may have limited generalizability owing to the inclusion of only surgical cases (both primary and additional surgical cases) as the gold standard for the reference standard for LNM and the exclusion of cases with endoscopic treatment alone. Specifically, patients with T1 CRC can undergo three types of treatment: (i) endoscopic treatment only; (ii) additional surgery after endoscopic treatment; and (iii) primary surgery. In clinical practice, additional staining should be considered for cases of endoscopic resection of T1 CRC (both endoscopic treatment only and additional surgery cases). The risk factors for LNM as indications for additional surgery are PD and LVI according to American guidelines, PD, LVI, and DSI according to European guidelines, and PD, LVI, DSI (greater than 1000 μm since 2005), and TB (since 2009) according to the Japanese guidelines.^3^, ^8^, ^11^, ^12^, ^13^, ^25^, ^26^ Notably, when all risk factors are absent, the likelihood of LNM is considered to be low because the guideline indications for LNM have high sensitivity.^100^ In the present study, the application of additional staining contributed to an elevated DOR for LNM, based on a multivariable meta‐analysis encompassing all four risk factors of PD, LVI, DSI, and TB. To achieve a comprehensive understanding of LNM in patients with T1 CRC, further studies encompassing cases treated exclusively with endoscopic treatment, which constitute ~30% of all T1 CRC,^20^ are essential.

In clinical practice, additional staining of D2‐40 for LI and elastic staining for VI should be performed when other risk factors for LNM are negative, because additional staining increases the sensitivity but does not change the specificity. Routine additional staining increases the detection rate for LI and VI.^101^, ^102^ In this review, we compared the diagnostic accuracy of different additional staining methods, such as EVG, VB, and CD31 staining, for VI. The additional staining for VI did not show significant differences between EVG and VB staining among elastic staining. However, CD31 staining for VI did not have sufficient data for evaluation because it was often assessed in conjunction with elastic staining,^37^, ^76^, ^83^ and only a few studies evaluated CD31 staining alone for VI.^38^, ^42^ In a previous study, CD31 staining was found to be less likely to detect VI than elastic staining and was not associated with prognosis.^103^ Further studies are needed to elucidate the optimal choice of staining methods and their appropriate utilization.

Our study has a few limitations. First, this review included mostly Asian studies and few European or North American studies, which may limit the generalizability. Further studies may determine the applicability of the present results to regions outside Asia. Second, we could not consider the cost of additional staining in this study. However, the introduction of additional staining into routine practice makes a small difference in cost ($4.97 per case or $44.75 per additional case of VI detection), which is insignificant when compared to the cost of adjuvant therapy, the correct utility of which depends on the accurate detection of LVI.^104^ Third, potential heterogeneity may exist in our analysis, although we used a model that accounted for the heterogeneity of within‐study and between‐study variation in the accuracy of study results.^28^ Potential sources of heterogeneity are that the evaluated patients differed among studies, resection specimens may be sectioned/handled differently (thereby, for instance, influencing the risk of retraction artifacts), histological assessment may be performed differently by pathologists with different levels of expertise or experience (e.g. general vs. dedicated gastrointestinal pathologist, local vs. centralized assessment), and the LVI criteria may differ. However, we performed sensitivity, subgroup, and meta‐regression analyses to assess as much as possible the potential sources of heterogeneity in this analysis.

In conclusion, the present systematic review and meta‐analysis showed that the DOR of additional staining for LNM may be higher, independent of other major risk factors (DSI, PD, and TB), than that of HE staining. Additional staining also may increase interobserver agreement among pathologists for LVI detection. These findings suggest that additional staining should be recommended to improve the criteria for secondary surgery following endoscopic resection when other risk factors for LNM are negative. Further studies are needed to validate the DOR of additional staining for LNM outside of Asia.

## CONFLICT OF INTEREST

Authors declare no conflict of interest for this article.

## FUNDING INFORMATION

This study was supported by JSPS KAKENHI (grant numbers JP21K21121 and 23K16289).

## Supporting information


**Appendix S1** PRISMA‐DTA.
**Appendix S2** Search strategy.
**Appendix S3** Amendments from the registered protocol.
**Appendix S4** The modified Quality Assessment of Diagnostic Accuracy Studies‐2 (QUADAS‐2) tool.
**Appendix S5** The risk of bias and applicability using the modified Quality Assessment of Diagnostic Accuracy Studies‐2 (QUADAS‐2) tool.
**Table S1** Overview of baseline characteristics.
**Table S2** Sensitivity analysis.
**Figure S1** Subgroup analysis of the diagnostic accuracy of (A) Elastica van Gieson (EVG) and (B) Victoria blue (VB) staining for vascular invasion on lymph node metastasis.
**Figure S2** Subgroup analysis of the diagnostic accuracy of hematoxylin–eosin (HE) and additional staining for lymphovascular invasion on lymph node metastasis in Japan and others.
**Figure S3** Subgroup analysis of the diagnostic accuracy of (A) D2‐40 staining for lymphatic invasion, (B) elastic staining for vascular invasion, and (C) additional staining for lymphovascular invasion on lymph node metastasis in (1) all or (2) limited cases.
