# Supplementary material for: Additional staining for lymphovascular invasion is associated with increased estimation of lymph node metastasis in patients with T1 colorectal cancer: Systematic review and meta‐analysis
Source: Dig Endosc. 2023 Oct 25;36(5):533–45. doi: 10.1111/den.14691 (PMC12136264; doi:10.1111/den.14691)
Supplement: Supplementary file 1 — Appendix S1 PRISMA‐DTA. Appendix S2 Search strategy. Appendix S3 Amendments from the registered protocol. Appendix S4 The modified Quality Assessment of Diagnostic Accuracy Studies‐2 (QUADAS‐2) tool. Appendix S5 The risk of bias and applicability using the modified Quality Assessment of Diagnostic Accuracy Studies‐2 (QUADAS‐2) tool. Table S1 Overview of baseline characteristics. Table S2 Sensitivity analysis. Figure S1 Subgroup analysis of the diagnostic accuracy of (A) Elastica van Gieson (EVG) and (B) Victoria blue (VB) staining for vascular invasion on lymph node metastasis. Figure S2 Subgroup analysis of the diagnostic accuracy of hematoxylin–eosin (HE) and additional staining for lymphovascular invasion on lymph node metastasis in Japan and others. Figure S3 Subgroup analysis of the diagnostic accuracy of (A) D2‐40 staining for lymphatic invasion, (B) elastic staining for vascular invasion, and (C) additional staining for lymphovascular invasion on lymph node metastasis in (1) all or (2) limited cases. [file DEN-36-533-s001.pdf]

Supplementary material Appendix S1: PRISMA-DTA

Section/topic	#	PRISMA-DTA Checklist Item	Reported on page #
**TITLE / ABSTRACT**			
Title	1	Identify the report as a systematic review (+/- meta-analysis) of diagnostic test accuracy (DTA) studies.	1
Abstract	2	Abstract: See PRISMA-DTA for abstracts.	4
**INTRODUCTION**			
Rationale	3	Describe the rationale for the review in the context of what is already known.	6
Clinical role of index test	D1	State the scientific and clinical background, including the intended use and clinical role of the index test, and if applicable, the rationale for minimally acceptable test accuracy (or minimum difference in accuracy for comparative design).	6, 7
Objectives	4	Provide an explicit statement of question(s) being addressed in terms of participants, index test(s), and target condition(s).	7
**METHODS**			
Protocol and registration	5	Indicate if a review protocol exists, if and where it can be accessed (e.g., Web address), and, if available, provide registration information including registration number.	7
Eligibility criteria	6	Specify study characteristics (participants, setting, index test(s), reference standard(s), target condition(s), and study design) and report characteristics (e.g., years considered, language, publication status) used as criteria for eligibility, giving rationale.	7-9
Information sources	7	Describe all information sources (e.g., databases with dates of coverage, contact with study authors to identify additional studies) in the search and date last searched.	7, 8
Search	8	Present full search strategies for all electronic databases and other sources searched, including any limits used, such that they could be repeated.	7, 8, Appendix B
Study selection	9	State the process for selecting studies (i.e., screening, eligibility, included in systematic review, and, if applicable, included in the meta-analysis).	8
Data collection process	10	Describe method of data extraction from reports (e.g., piloted forms, independently, in duplicate) and any processes for obtaining and confirming data from investigators.	8, 9
Definitions for data extraction	11	Provide definitions used in data extraction and classifications of target condition(s), index test(s), reference standard(s) and other characteristics (e.g. study design, clinical setting).	8, 9

Risk of bias and applicability	12	Describe methods used for assessing risk of bias in individual studies and concerns regarding the applicability to the review question.	8, 9
Diagnostic accuracy measures	13	State the principal diagnostic accuracy measure(s) reported (e.g. sensitivity, specificity) and state the unit of assessment (e.g. per-patient, per-lesion).	9, 10
Synthesis of results	14	Describe methods of handling data, combining results of studies and describing variability between studies. This could include, but is not limited to: a) handling of multiple definitions of target condition. b) handling of multiple thresholds of test positivity, c) handling multiple index test readers, d) handling of indeterminate test results, e) grouping and comparing tests, f) handling of different reference standards	9, 10
Meta-analysis	D2	Report the statistical methods used for meta-analyses, if performed.	9, 10
Additional analyses	16	Describe methods of additional analyses (e.g., sensitivity or subgroup analyses, meta-regression), if done, indicating which were pre-specified.	10, 11
**RESULTS**			
Study selection	17	Provide numbers of studies screened, assessed for eligibility, included in the review (and included in meta-analysis, if applicable) with reasons for exclusions at each stage, ideally with a flow diagram.	11, Figure 1
Study characteristics	18	For each included study provide citations and present key characteristics including: a) participant characteristics (presentation, prior testing), b) clinical setting, c) study design, d) target condition definition, e) index test, f) reference standard, g) sample size, h) funding sources	11, Supplementary Table 1
Risk of bias and applicability	19	Present evaluation of risk of bias and concerns regarding applicability for each study.	11, 12, Appendix D
Results of individual studies	20	For each analysis in each study (e.g. unique combination of index test, reference standard, and positivity threshold) report 2x2 data (TP, FP, FN, TN) with estimates of diagnostic accuracy and confidence intervals, ideally with a forest or receiver operator characteristic (ROC) plot.	12–14
Synthesis of results	21	Describe test accuracy, including variability; if meta-analysis was done, include results and confidence intervals.	12–14
Additional analysis	23	Give results of additional analyses, if done (e.g., sensitivity or subgroup analyses, meta-regression; analysis of index test: failure rates, proportion of inconclusive results, adverse events).	12–14
**DISCUSSION**			

Summary of evidence	24	Summarize the main findings including the strength of evidence.	14, 15
Limitations	25	Discuss limitations from included studies (e.g. risk of bias and concerns regarding applicability) and from the review process (e.g. incomplete retrieval of identified research).	18, 19
Conclusions	26	Provide a general interpretation of the results in the context of other evidence. Discuss implications for future research and clinical practice (e.g. the intended use and clinical role of the index test).	19
**FUNDING**			
Funding	27	For the systematic review, describe the sources of funding and other support and the role of the funders.	20

Supplementary material Appendix S2: Search strategy

The following search strategy was used form inception until December 10, 2022.

CENTRAL

#1. [mh "Colorectal Neoplasms"]

#2. ((colorect*:ti,ab OR colon*:ti,ab OR rect*:ti,ab) NEAR/1 (carcinoma*:ti,ab OR neoplas*:ti,ab OR adenocarcinom*:ti,ab OR cancer*:ti,ab OR tumor*:ti,ab OR tumour*:ti,ab OR malignan*:ti,ab))

#3. #1 OR #2

#4. (T1:ti,ab OR pT1:ti,ab OR submucosal:ti,ab OR early:ti,ab)

#5. #3 AND #4

#6. ("Victoria blue":ti,ab OR "Elastica van Gieson":ti,ab OR EVG:ti,ab OR “D2-40”:ti,ab OR podoplanin:ti,ab)

#7. ((lymph*:ti,ab OR vascular*:ti,ab OR lymphovascular*:ti,ab) NEAR/1 invasion*:ti,ab)

#8. #6 OR #7

#9. #5 AND #8

MEDLINE (via Ovid)

1 exp Colorectal Neoplasms/

2 ((colorect* or colon* or rect*) adj1 (carcinoma* or neoplas* or adenocarcinom* or cancer* or tumor* or tumour* or malignan*)).tw.

3 1 or 2

4 (T1 or pT1 or submucosal or early).tw.

5 3 and 4

6 ("Victoria blue" or "Elastica van Gieson" or EVG or D2-40 or podoplanin).tw.

7 ((lymph* or vascular* or lymphovascular*) adj1 invasion*).tw.

8 6 or 7

9 5 and 8

EMBASE (via ProQuest Dialog)

S1 (EMB.EXACT.EXPLODE("colorectal tumor"))

S2 ((ti(colorect*) or ti(colon*) or ti(rect*)) AND (ti(carcinoma*) or ti(neoplas*) or ti(adenocarcinom*) or ti(cancer*) or ti(tumor*) or ti(tumour*) or ti(malignan*))) OR ((ab(colorect*) or ab(colon*) or ab(rect*)) AND (ab(carcinoma*) or ab(neoplas*) or ab(adenocarcinom*) or ab(cancer*) or ab(tumor*) or ab(tumour*) or ab(malignan*)))

S3 S1 OR S2

S4 (ti(T1) or ti(pT1) or ti(submucosal) or ti(early)) OR (ab(T1) or ab(pT1) or ab(submucosal) or ab(early))
S5 S3 AND S4
S6 (ti("Victoria blue") or ti("Elastica van Gieson") or ti(EVG) or ti(D2-40) or ti(podoplanin)) OR (ab("Victoria blue") or ab("Elastica van Gieson") or ab(EVG) or ab(D2-40) or ab(podoplanin))
S7 ((ti(lymph*) or ti(vascular*) or ti(lymphovascular*)) AND ti(invasion*)) OR ((ab(lymph*) or ab(vascular*) or ab(lymphovascular*)) AND ab(invasion*))
S8 S6 OR S7
S9 S5 AND S8

ICTRP

((colorectal or colon or rectum) AND (carcinoma or carcinomas or neoplasm or neoplasms or adenocarcinoma or adenocarcinomas or cancer or cancers or tumor or tumors or tumour or tumours or malignancy or malignancies)) AND (T1 or pT1 or submucosal or early)

ClinicalTrials.gov

("Victoria blue" or "Elastica van Gieson" or EVG or D2-40 or podoplanin) OR ((lymph or vascular or lymphovascular) AND invasion)

Supplementary material Appendix S3: Amendments from the registered protocol

The protocol planned a subgroup analysis of primary and additional surgical resection but was changed to a sensitivity analysis that excluded studies with endoscopic resection cases. We also added the sensitivity analysis of only studies with primary surgical resection. Additionally, we added the subgroup analysis of only studies that compared additional staining with HE staining in the same patients or institution. We added the subgroup analysis of additional staining indications (limited cases vs. all cases) and sensitivity- and specificity analysis of CD31 staining for VI. We could not perform the prespecified sensitivity analysis of only studies without missing data because of no studies with missing data.

Supplementary material Appendix S4: The modified Quality Assessment of Diagnostic Accuracy Studies-2 (QUADAS-2) tool

Risk of Bias

Patient Selection

Was a consecutive or random sample of patients enrolled?

Was a case–control design avoided?

Did the study avoid inappropriate exclusions?

We will judge participants based on our inclusion/exclusion criteria.

Index Text

Were the index test results interpreted without knowledge of the results of the reference standard?

Reference Standard

Is the reference standard likely to correctly classify the target condition?

We will judge D0 dissection surgery inappropriate.

Were the reference standard results interpreted without knowledge of the results of the index test?

Flow and Timing

Was there an appropriate interval between the index test and reference standard?

Did all patients receive the same reference standard?

Were all patients included in the analysis?

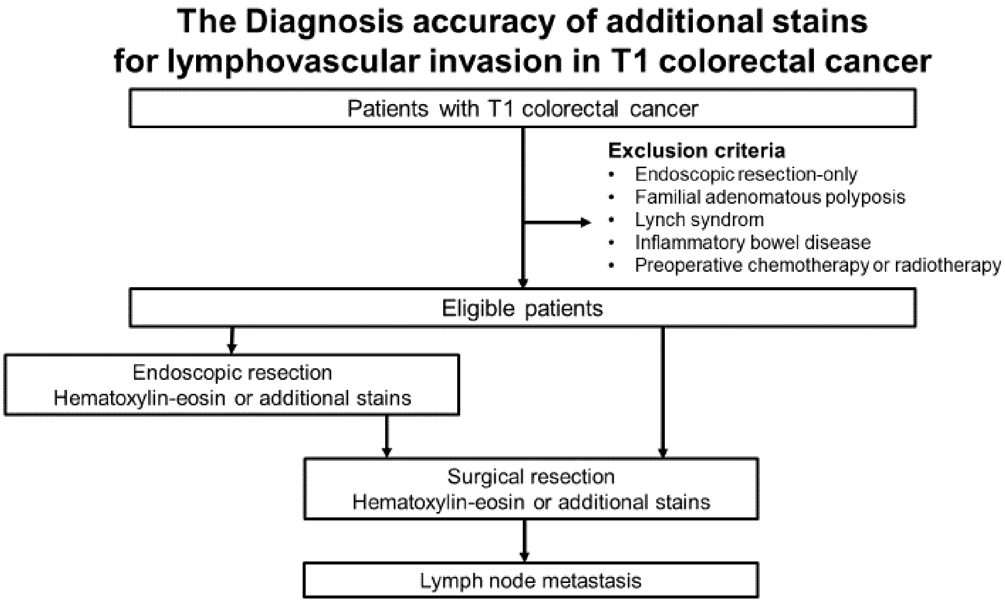

Applicability

Patient Selection

Are There Concerns That the Included Patients and Setting Do Not Match the Review Question?

Index Test

Are There Concerns That the Index Test, Its Conduct, or Its Interpretation Differ From the Review Question?

Reference Standard

Are There Concerns That the Target Condition as Defined by the Reference Standard Does Not Match the Question?

Supplementary material Appendix S5: The risk of bias and applicability using the modified Quality Assessment of Diagnostic Accuracy Studies-2 (QUADAS-2) tool

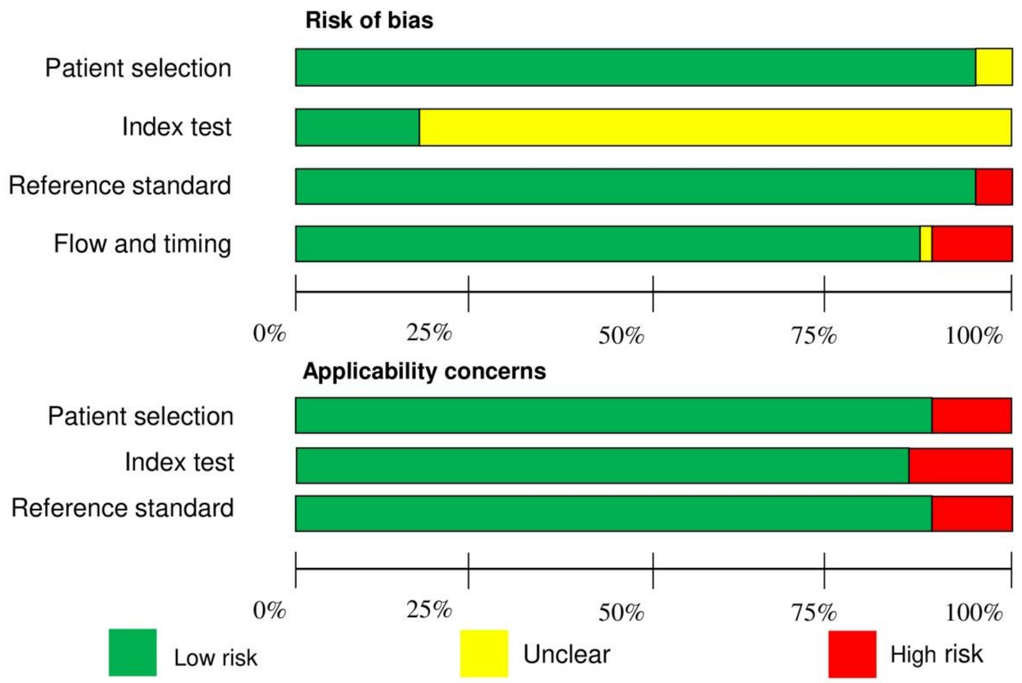

Authors, year	Risk of bias				Applicability concerns		
	Patient selection	Index test	Reference standard	Flow and timing	Patient selection	Index test	Reference standard
Ebbehøj, 2023	Low	Unclear	Low	Low	Low	Low	Low
Bae, 2022	Low	Unclear	Low	Low	Low	High	Low
Cho, 2022	Low	Unclear	Low	High	High	High	High
Gambella, 2022	Low	Unclear	Low	Low	Low	Low	Low
Kim, 2022	Low	Unclear	Low	Low	Low	Low	Low
Morini, 2022	Low	Low	Low	Low	Low	Low	Low
Ozeki, 2022	Low	Unclear	Low	Low	Low	High	Low
Ronnow, 2022	Low	Unclear	Low	Low	Low	Low	Low
Song, 2022	Low	Low	Low	Low	Low	Low	Low
Cheng, 2021	Low	Low	Low	Low	Low	Low	Low
Kang, 2021	Low	Unclear	Low	Low	Low	Low	Low
Naito, 2021	Low	Unclear	Low	Low	Low	Low	Low
Nishimura, 2021	Low	Low	Low	Low	Low	Low	Low
Sugai, 2021	Unclear	Unclear	Low	Unclear	Low	Low	Low
Takashina, 2021	Low	Unclear	Low	High	High	Low	High
Dou, 2020	Low	Low	Low	High	High	Low	High
Mochizuki, 2020	Low	Unclear	Low	Low	Low	Low	Low
Haasnoot, 2020	Low	Unclear	Low	Low	Low	Low	Low
Yamaoka, 2020	Low	Unclear	Low	Low	Low	Low	Low
Barel, 2019	Low	Unclear	High	High	High	High	High
Kishida, 2019	Low	Unclear	Low	Low	Low	Low	Low
Makimoto, 2019	Low	Low	Low	Low	Low	Low	Low
Takamatsu, 2019	Low	Unclear	High	High	High	Low	High
Yasue, 2019	Low	Unclear	Low	Low	Low	Low	Low
Yoshizumi, 2019	Low	Unclear	Low	Low	Low	Low	Low
Zhang, 2019	Low	Unclear	Low	Low	Low	Low	Low
Chernyshov, 2018	Low	Unclear	Low	Low	Low	Low	Low
Han, 2018	Low	Unclear	Low	Low	Low	Low	Low
Lee, 2018	Low	Unclear	Low	Low	Low	Low	Low
Yim, 2017	Low	Unclear	Low	Low	Low	High	Low
Machado, 2016	Low	Unclear	High	High	High	Low	High
Kawachi, 2015	Low	Unclear	Low	Low	Low	Low	Low
Toh, 2015	Low	Unclear	Low	Low	Low	Low	Low

Barresi, 2014	Unclear	Unclear	Low	Unclear	Low	Low	Low
Lee, 2014	Low	Unclear	Low	Low	Low	High	Low
Nishida, 2014	Low	Unclear	Low	Low	Low	Low	Low
Suh, 2013	Low	Low	Low	Low	Low	Low	Low
Wada, 2013	Low	Unclear	Low	Low	Low	High	Low
Nakadoi, 2012	Low	Unclear	Low	Low	Low	Low	Low
Akishima-Fukasawa, 2011	Low	Unclear	Low	Low	Low	High	Low
Ishii, 2010	Low	Unclear	Low	Low	Low	High	Low
Komori, 2010	Low	Unclear	Low	Low	Low	Low	Low
Tateishi, 2010	Low	Unclear	Low	Low	Low	Low	Low
Suzuki, 2009	Low	Unclear	Low	Low	Low	Low	Low
Kawaura, 2007	Low	Unclear	Low	Low	Low	Low	Low
Nakajo, 2007	Unclear	Unclear	Low	Low	Low	Low	Low
Yasuda, 2007	Low	Unclear	Low	Low	Low	High	Low
Kazama, 2006	Low	Unclear	Low	Low	Low	Low	Low
Wang, 2005	Low	Unclear	Low	Low	Low	Low	Low
Watanabe, 2005	Low	Unclear	Low	Low	Low	Low	Low
Kitajima, 2004	Low	Unclear	Low	Low	Low	Low	Low
Okabe, 2004	Low	Unclear	Low	Low	Low	Low	Low
Taira, 2004	Low	Low	Low	Low	Low	Low	Low
Ueno, 2004	Low	Unclear	Low	Low	Low	High	Low
Asai, 2003	Low	Unclear	Low	Low	Low	Low	Low
Sakuragi, 2003	Low	Unclear	Low	High	High	Low	High
Suzuki, 2003	Low	Unclear	Low	Low	Low	Low	Low
Tsuruta, 2000	Low	Unclear	Low	Low	Low	Low	Low
Inoue, 1999	Low	Unclear	Low	Low	Low	Low	Low
Coverlizza, 1989	Low	Low	Low	Low	Low	Low	Low

Risk of bias and applicability are rated on seven domains using the modified Quality Assessment of Diagnostic Accuracy Studies-2 tool by two of the four reviewers (JW and MA, HS, or MH). The four domains assessed for bias are “Patient selection,” “Index test,” “Reference standard,” and “Flow and timing,” and the three domains assessed for applicability are “Patient selection,” “Index test,” and “Reference standard.” Each risk of bias and applicability domain is classified as high, low, and unclear according to prespecified signaling questions in protocol (https://osf.io/6f5qt/).

Supplementary Table S1. Overview of baseline characteristics

Authors, year	Country	Study design	Recruitment period	Total patients, n	Male, n	Age (years)	Total number of LNM, n (%)	Stains (lymphatic/ vascular invasion)	Indication for additional stains	Treatment method (%)	Indication for surgery	Definition of lymphovascular invasion
Ebbehøj, 2023	Denmark	Retrospective study	2016-2019	1167	639	68	170 (14.6)	HE	NA	Primary and additional surgery (NA)	ASCRS, ESGE, ESMO, and/or JSCCR guidelines	LVI is evaluated according to the WHO classification. VI comprises both intramural and extramural invasion.
Bae, 2022	Korea	Retrospective study	2000-2015	277	148	60	30 (10.8)	HE/ EVG, CD31	All	Primary (70%) and additional surgery (30%)	JSCCR guidelines	LI and VI are evaluated according to the JSCCR classification 2019.
Cho, 2022	Korea	Retrospective study	2010-2020	380	232	66	37 (9.7)	D2-40/ CD31	If needed	Primary (46%) and additional surgery (49%), and endoscopy (5%)	PD, DPI, LI, VI, perineural invasion, and positive margins	LI and VI are defined as the presence of lymphatic and vascular vessels and tumor cells.
Gambella, 2022	Italy	Retrospective study	2010-2019	207	93	70	18 (8.7)	HE	NA	Primary and additional surgery (NA)	NA	LVI is evaluated according to the WHO classification 5th edition. LVI is reported when tumor cells are identified within endothelial-outlined peritumoral stromal vessels.

Kim, 2022	Korea	Retrospective study	2002-2019	395	225	63	52 (13.2)	HE	NA	Primary and additional surgery (NA)	NA	LVI is evaluated according to the Korean Society of Pathologists classification. LI or VI is considered present when tumor cells invade non-muscle-walled small vessels or large vessels with a smooth muscle layer and/or an elastic lamina layer.
Morini, 2022	Italy	Retrospective study	2000-2018	122	65	NA	15 (12.3)	HE	NA	Additional surgery (100%)	AJCC	LVI was assessed according to the guidelines of this College of American Pathologists.
Ozeki, 2022	Japan	Retrospective study	2003-2019	285	154	69	32 (11.2)	D2-40/ HE, CD31	If needed	Primary and additional surgery (NA)	JSCCR guidelines 2019	LI and VI are evaluated according to the JSCCR classification 2019.
Ronnow, 2022	Sweden/ Denmark	Prospective study	2009-2017/ 2016-2018	1439	752	71	150 (10.4)	HE	NA	Primary and additional surgery (NA)	NA	LVI is evaluated for both intramural and extramural vascular invasion as well as lymphatic invasion.
Song, 2022	Korea	Retrospective study	2010-2018	400	239	59	71 (17.8)	D2-40	If needed	Additional surgery (100%)	NA	LI and VI are evaluated according to the JSCCR classification 2016.

Cheng, 2021	China	Retrospective study	2015-2019	62	39	58	12 (19.4)	D2-40/ VB	All	Additional surgery (100%)	JSCCR guidelines 2016	LI and VI are evaluated according to the JSCCR classification 2016.
Kang, 2021	Korea	Retrospective study	2004-2011	221	123	NA	29 (13.1)	HE	NA	Primary and additional surgery (NA)	PD, DPI, LVI, and positive margins	NA
Naito, 2021	Japan	Retrospective study	2008-2018	239	152	67	27 (11.3)	HE/ VB	Unknown	Primary (59%) and additional surgery (41%)	JSCCR guidelines	NA
Nishimura, 2021	Japan	Prospective study	2012-2017	215	NA	NA	21 (9.8)	D2-40/ VB, EVG	All	Additional surgery (100%)	JSCCR guidelines	LI and VI are evaluated according to the JSCCR classification 2016.
Sugai, 2021	Japan	Retrospective study	NA	115	NA	NA	37 (32.2)	D2-40/ EVG	All	Primary and additional surgery (NA)	JSCCR guidelines 2016	LI and VI are evaluated according to the JSCCR classification 2016.
Takashina, 2021	Japan	Retrospective study	2005-2012	1152	729	66	88 (11)*	D2-40/ VB	All	Primary and additional surgery (69%), and endoscopy (31%)	JSCCR guidelines	LI and VI are evaluated according to the JSCCR classification 2019.
Dou, 2020	China	Retrospective study	2008-2016	550	270	60	4 (13.3)*	HE	NA	Additional surgery (5%) and endoscopy (95%)	China guideline 2015	NA

Mochizuki, 2020	Japan	Retrospective study	2001-2018	745	460	66	75 (10.1)	D2-40/ VB	All	Primary (54%) and additional surgery (46%)	JSCCR guidelines	LI and VI are evaluated according to the JSCCR classification 2019.
Haasnoot, 2020	Netherlands	Retrospective study, case-cohort	2000-2014	225	NA	NA	43 (19.1)	HE	NA	Primary and additional surgery (NA)	NA	LVI is defined as the presence of cancer cells within endothelial-lined channels.
Yamaoka, 2020	Japan	Retrospective study	2002-2012	548	335	66	58 (10.6)	D2-40/ EVG	If needed	Primary (55%) and additional surgery (45%)	JSCCR guidelines	LI and VI are evaluated according to the JSCCR classification 2019.
Barel, 2019	France	Retrospective study	2009-2013	234	134	67	19 (8.1)	Cytokeratin, D2-40/ HE, CD31	All	Primary and additional surgery, and endoscopy (NA)	JSCCR or ESGE guidelines	LI is diagnosed in cases in which cancer cells are seen within endothelia cell-lined small vessels and VI when tumor cells are seen in the lumen of large vessels with a muscle layer.
Kishida, 2019	Japan	Retrospective study	2014-2017	219	127	68	34 (15.5)	D2-40/ EVG	All	Primary and additional surgery (NA)	JSCCR guidelines	LI and VI are evaluated according to the JSCCR classification 2014.
Makimoto, 2019	Japan	Retrospective study	2010-2018	53	24	68	8 (15.1)	HE	NA	Additional surgery (100%)	JSCCR guidelines	LI and VI are evaluated according to the JSCCR classification 2010 and 2016.

Takamatsu, 2019	Japan	Retrospective study	2005-2012	318	182	62	27 (8.5)	HE/ VB	All	Primary and additional surgery, and endoscopy (NA)	JSCCR guidelines	LI and VI are evaluated according to the JSCCR classification 2014.
Yasue, 2019	Japan	Retrospective study	2005-2016	846	470	66	74 (8.7)	D2-40/ VB	SM invasive cancer is diag-nosed using HE-stained specimens.	Primary and additional surgery, and endoscopy (NA)	JSCCR guidelines	LI and VI are evaluated according to the JSCCR classification 2020.
Yoshizumi, 2019	Japan	Retrospective study	2010-2017	118	81	69	12 (9.0)	D2-40/ EVG	All	Primary (69%) and additional surgery (31%)	JSCCR guidelines 2016	LI and VI are evaluated according to the JSCCR classification 2016.
Zhang, 2019	China	Retrospective study	2008-2014	290	151	60	45 (15.5)	D2-40/ EVG	Suspicious areas of LVI	Primary and additional surgery (NA)	NA	LVI is evaluated according to the WHO classification 4th edition.
Chernyshov, 2018	Russia	Retrospective study	2012-2018	53	19	62	11 (20.8)	HE	NA	Primary surgery (100%)	AJCC	NA

Han, 2018	Korea	Retrospective study	2008-2012	492	296	61	55 (11.2)	HE	NA	Primary (48%) and additional surgery (52%)	NA	LI and VI are evaluated according to the JSCCR classification 2010.
Lee, 2018	Korea	Retrospective study	2010-2016	133	83	63	16 (12.0)	HE	NA	Primary (35%) and additional surgery (65%)	NA	LVI is evaluated according to the JSCCR classification 2014.
Yim, 2017	Korea	Retrospective study	2000-2015	252	130	61	31 (12.3)	D2-40/ HE, CD31, CD34	It is difficult to judge the presence or absence of lymphovascular invasion.	Primary surgery (100%)	NA	LI and VI is evaluated according to the JSCCR classification 2010.
Machado, 2016	Spain	Retrospective study	2006-2014	29	19	63	5 (17.2)	HE	NA	Primary (28%) and additional surgery (72%)	NA	NA
Kawachi, 2015	Japan	Retrospective study	1976-2007	806	482	64	97 (12.0)	HE	NA	Primary and additional surgery (NA)	JSCCR guidelines	LI and VI are evaluated according to the JSCCR classification 2010.

Toh, 2015	United Kingdom	Prospective study	NA	207	127	71	19 (9.2)	HE	NA	Additional surgery and endoscopy (NA)	NA	NA
Barresi, 2014	Italy	Retrospective study	NA	101	59	70	8 (7.9)	HE, D2-40	All	Primary surgery (100%)	NA	LVI is evaluated according to the JSCCR classification 2010.
Lee, 2014	Korea	Retrospective study	2000-2010	263	NA	NA	31 (11.8)	HE/CD34	Selected cases	Primary and additional surgery (NA)	NA	NA
Nishida, 2014	Japan	Retrospective study	2000-2011	265	161	65	31 (11.7)	D2-40/ EVG	All	Primary surgery (100%)	JSCCR guidelines 2010	LI and VI are evaluated according to the JSCCR classification 2010.
Suh, 2013	Korea	Prospective study	2007-2012	75	NA	NA	10 (13.3)	HE	NA	Additional surgery (100%)	NA	NA
Wada, 2013	Japan	Retrospective study	1995-2005	120	82	65	12 (10)	D2-40/ vWF	All	Primary (63%) and additional surgery (37%)	JSCCR guidelines	NA
Nakadoi, 2012	Japan	Retrospective study	1981-2008	499	NA	63	41 (8.2)	HE	NA	Primary (74%) and additional surgery (26%)	JSCCR guidelines	LI and VI are evaluated according to the JSCCR classification 2010.
Akishima-	Japan	Retrospective study	1989-2009	111	76	67	36 (32.4)	LYVE-1/ VB, vWF	All	Primary and additional surgery (NA)	JSCCR guidelines	Cancer cells in a LYVE-1-positive vessel structure are evaluated as LI. Cancer cells

Fukasawa, 2011												in VB and/or vWF-positive vascular structures are evaluated as VI.
Ishii, 2010	Japan	Retrospective study	25 years	203	NA	NA	16 (7.9)	HE, D2-40/ HE, EVG, CD31, CD34	All	Primary and additional surgery (NA)	JSCCR guidelines	Cancer cells in a D2-40-positive vessel structure are evaluated as LI. Cancer cells in EVG, CD31 and/ or CD34-positive vascular structures are evaluated as VI.
Komori, 2010	Japan	Retrospective study	1990-2004	111	59	61	17 (15.3)	HE	NA	Primary and additional surgery (NA)	JSCCR guidelines	NA
Tateishi, 2010	Japan	Retrospective study	1992-2005	322	216	61	46 (14.3)	HE	NA	Primary surgery (100%)	JSCCR guidelines	NA
Suzuki, 2009	Japan	Retrospective study	1990-2004	124	85	64	18 (14.5)	D2-40/ EVG	All	Primary and additional surgery (NA)	JSCCR guidelines	LI and VI are evaluated according to the JSCCR classification 2010.
Kawaura, 2007	Japan	Retrospective study	1992-2005	122	77	62	20 (16.4)	HE, D2-40/ EVG	All	Primary surgery (100%)	JSCCR guidelines	One slice of the same section is used for Elastica van Gieson staining to examine the tumor tissue for VI by tumor cells, and another slice is used for

												immunohistochemical staining with D2-40 monoclonal antibody to detect LI by tumor cells.
Nakajo, 2007	Japan	Retrospective study	1985-2005	283	182	66	20 (7.1)	HE	NA	Primary (57%) and additional surgery (43%)	JSCCR guidelines	NA
Yasuda, 2007	Japan	Retrospective study	NA	86	57	66	21 (24.4)	VB, CD31	All	Primary surgery (100%)	JSCCR guidelines	LVI is regarded as cancerous involvement of lymphatic and/or vascular vessels.
Kazama, 2006	Japan	Retrospective study	1990-2001	56	41	63	16 (28.6)	D2-40/ VB	All	Primary surgery (100%)	JSCCR guidelines	NA
Wang, 2005	Taiwan	Retrospective study	1969-2002	159	107	65	16 (10.1)	HE	NA	Primary and additional surgery (NA)	NA	LI is defined as the presence of tumor within spaces surrounded by a clearly visualized endothelial lining. VI is documented if the presence of additional fibrin clots, erythrocytes, or both are observed in an endothelial-lined space without erythrocyte extravasation into the

												surrounding tissue or by evidence of tumor within a smooth muscle-lined space.
Watanabe, 2005	Japan	Retrospective study	1997-2003	59	36	65	9 (15.3)	HE	NA	Primary surgery (100%)	JSCCR guidelines	NA
Kitajima, 2004	Japan	Retrospective study	NA	865	514	63	87 (10.1)	HE	NA	Primary and additional surgery (NA)	JSCCR guidelines	NA
Okabe, 2004	Japan/ USA	Retrospective study	1987-2001	428	258	64	41 (9.6)	HE	NA	Primary surgery (100%)	NA	NA
Taira, 2004	Japan	Retrospective study	1973-2000	115	96	58	6 (5.2)	EVG	Unknown	Additional surgery (100%)	JSCCR guidelines	NA
Ueno, 2004	Japan	Retrospective study	1980-2002	251	147	62	33 (13.1)	HE/ EVG, CD34	Paraffin-embedded tumors were available (53%)	Primary (68%) and additional surgery (32%)	JSCCR guidelines	LI and VI are defined as cancer involvement of lymphatic vessels and/or vascular vessels.
Asai, 2003	Japan	Retrospective study	1987-2001	113	72	60	14 (12.4)	HE/ EVG	If needed	Primary (66%) and additional surgery (34%)	JSCCR guidelines	LI is judged as positive only when the lymphatic vessels covered with endothelial cells are found to be infiltrated with cancer on HE staining.

												VI is judged as positive only when HE staining or EVG staining clearly shows cancerous invasion into the veins.
Sakuragi, 2003	Japan	Retrospective study	1979-2000	271	175	62	21 (7.7)	HE	NA	Primary (48%) and additional surgery (36%), and endoscopy (16%)	JSCCR guidelines	LI is defined as the presence of tumor cells within small luminal structures lined by endothelial cells. Lymphatic vessels are distinguished from blood vessels by the presence of luminal red blood cells or from the vessel wall morphology (i.e., the presence of smooth muscle).
Suzuki, 2003	Japan	Retrospective study	1975-2000	124	NA	63	18 (14.5)	HE/ VB	If needed	Primary surgery (100%)	JSCCR guidelines	LI is defined as the presence of tumor cells within small luminal structures lined by endothelial cells. Lymphatic vessels are distinguished from blood vessels by the presence of luminal red blood cells or from the vessel wall

												morphology (i.e., the presence of smooth muscle).
Tsuruta, 2000	Japan	Retrospective study	1995-1999	77	56	63	13 (16.9)	HE/ EVG	All	Primary (75%) and additional surgery (25%)	JSCCR guidelines	LI is determined by the presence of tumor cells in the lumen covered with endothelial cells in the absence of erythrocytes. VI is determined by the presence of tumor cells in the lumen where endothelial cells covered with the smooth muscle with elastic laminae, as detected by EVG staining, are present.
Inoue, 1999	Japan	Retrospective study	10 years	86	NA	NA	11 (12.8)	HE	NA	Primary surgery (100%)	JSCCR guidelines	LI and VI are evaluated according to the JSCCR classification 1994.
Coverliza, 1989	Italy	Retrospective study	1975-1987	14	7	57	5 (35.7)	HE	NA	Additional surgery (100%)	NA	The presence or absence of VI is determined only after an average of 20 sections per case are examined. VI is considered to occur only when both pathologists are in

agreement on this feature.

Doubtful cases are excluded.

*Only cases with radical surgery or completion surgery are included. If endoscopic resection is performed without completion surgery and no specific statement is reported about LNM status, cases are excluded.

Abbreviations used in this paper: AJCC, American Joint Commission on Cancer; ASCRS, American Society of Colon and Rectal Surgeons; CI confidence interval; ESGE, European Society of Gastrointestinal Endoscopy; ESMO, European Society for Medical Oncology; EVG, Elastica van Gieson; DSI, deep submucosal invasion; HE, hematoxylin-eosin; JCCRS, Japanese Society for Cancer of the Colon and Rectum; LNM, lymph node metastasis; LI, lymphatic invasion; LVI, lymphatic and/or vascular invasion; NA, not applicable; PD, poorly differentiation; VB, Victoria blue; VI, vascular invasion; WHO, World Health Organization

Supplementary Figure S1. Subgroup analysis of the diagnostic accuracy of (A) EVG and (B) VB staining for vascular invasion on lymph node metastasis.

(A) EVG staining for VI

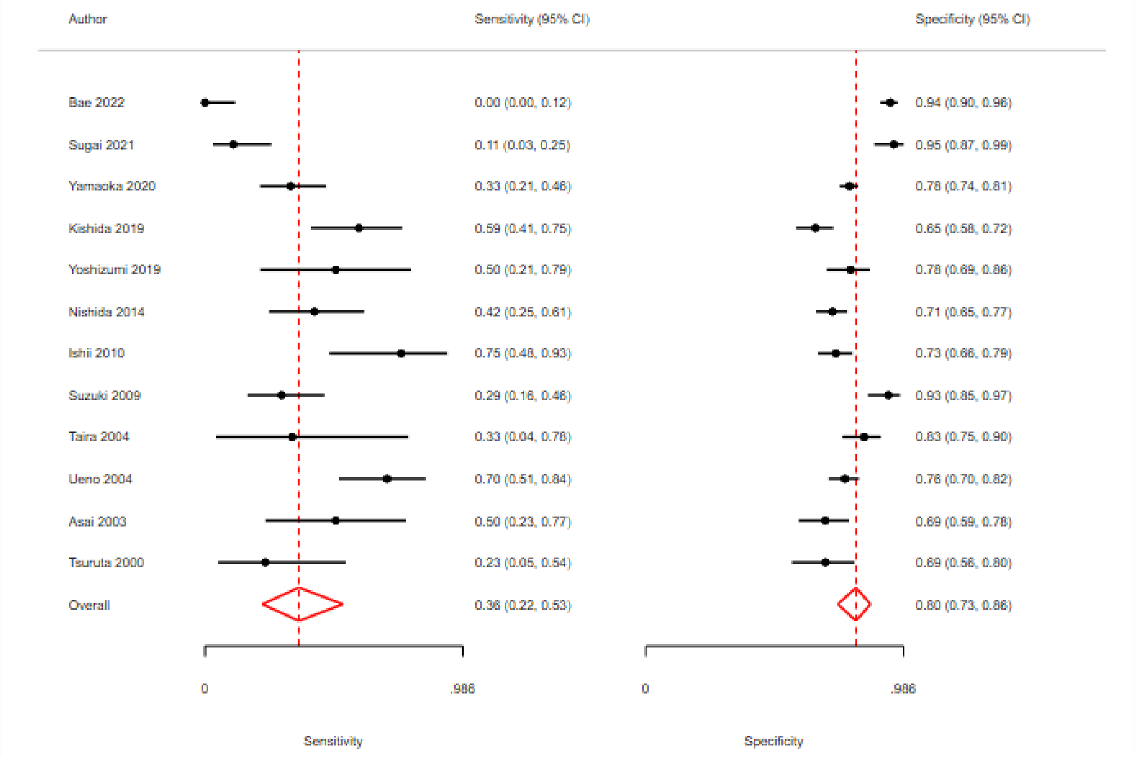

(B) VB staining for VI

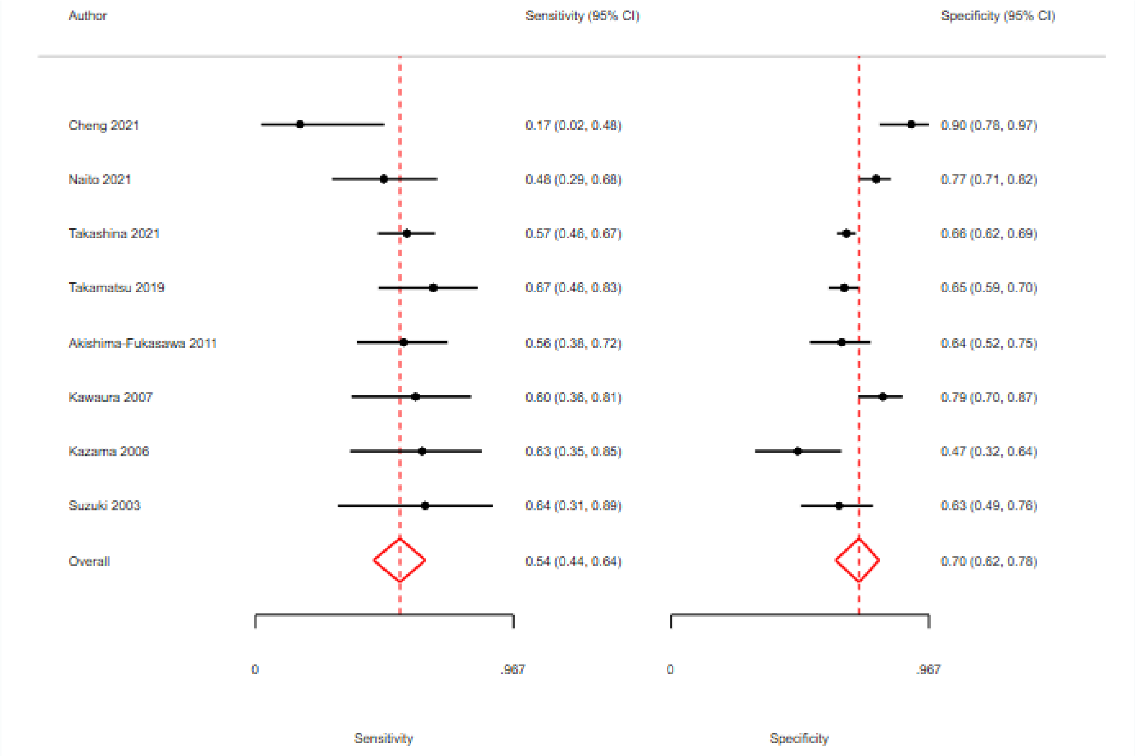

Supplementary Figure S2. Subgroup analysis of the diagnostic accuracy of HE and additional staining for lymphovascular invasion on lymph node metastasis in Japan and other countries.

(A) HE stains for LVI in Japan

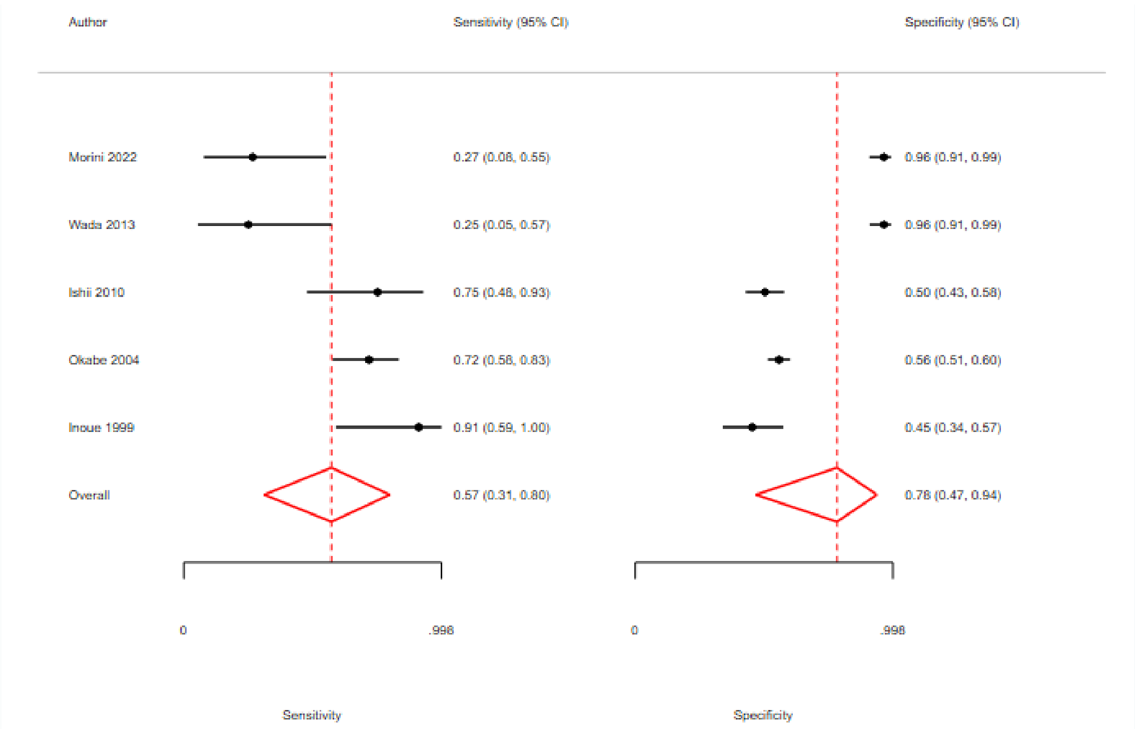

(B) HE stains for LVI in other countries

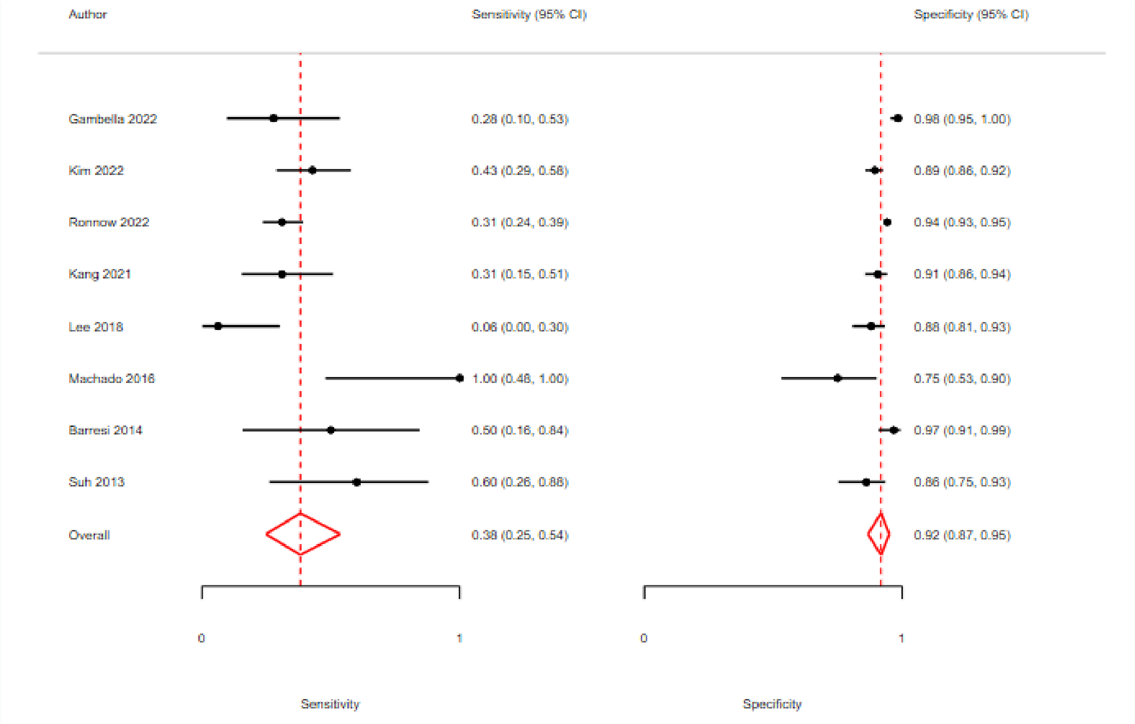

(C) Additional staining for LVI in Japan

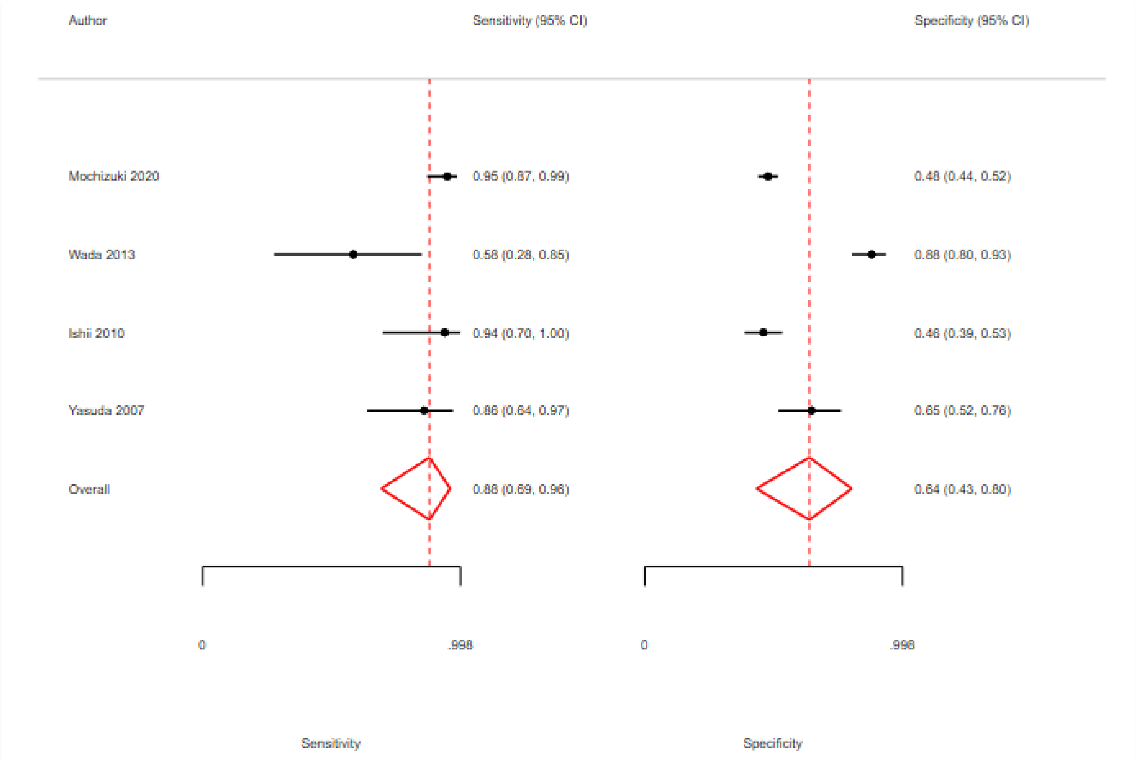

(D) Additional staining for LVI in other countries

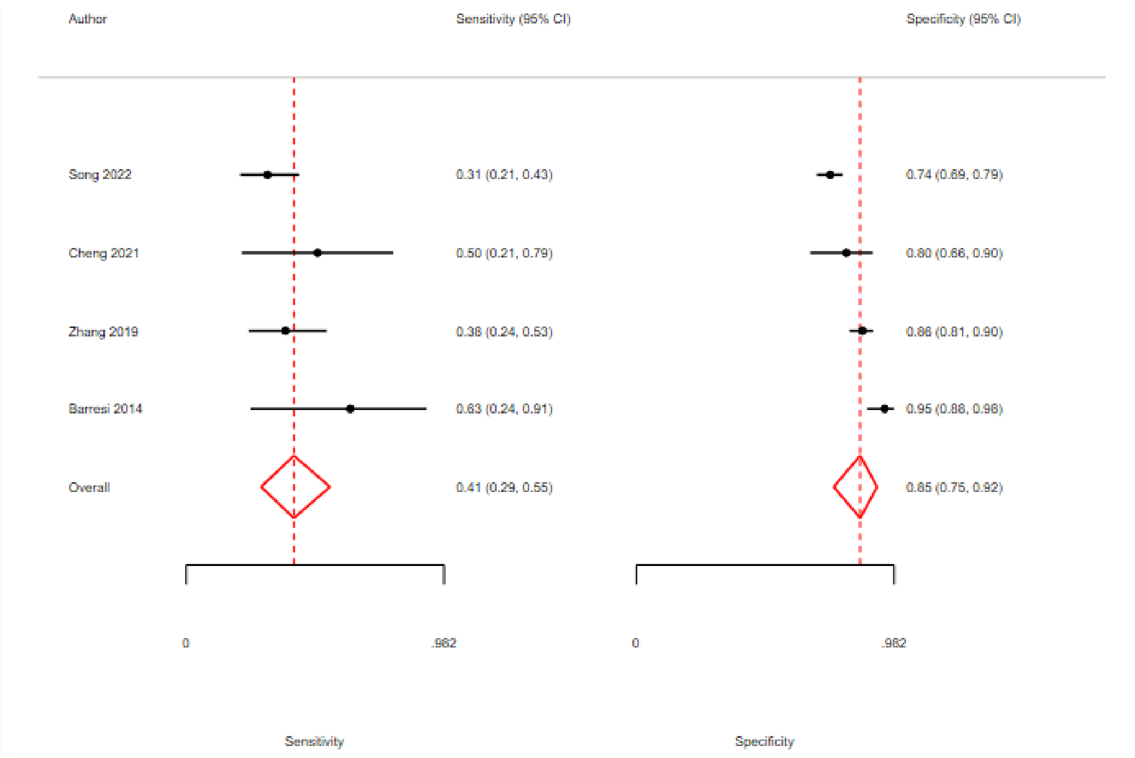

Supplementary Figure S3. Subgroup analysis of the diagnostic accuracy of (A) D2-40 staining for lymphatic invasion, (B) elastic staining for vascular invasion, and (C) additional staining for lymphovascular invasion on lymph node metastasis in (1) all or (2) limited cases.

(A1) D2-40 staining for LI in all cases

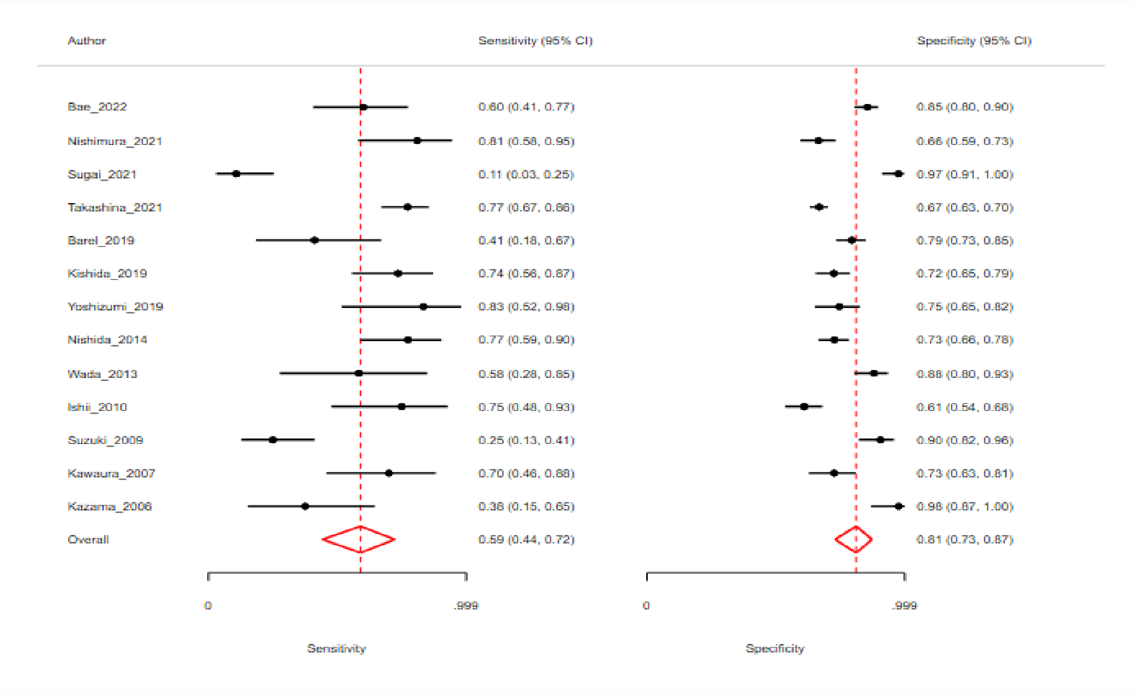

(A2) D2-40 staining for LI in limited cases

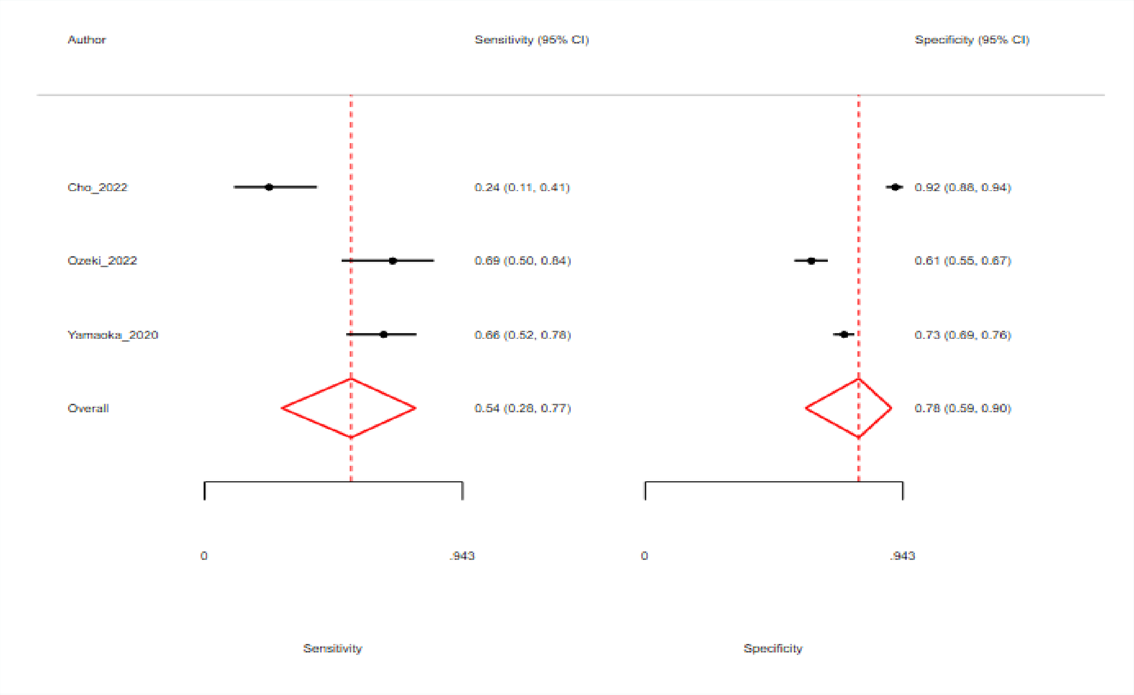

(B1) Elastic staining for VI in all cases

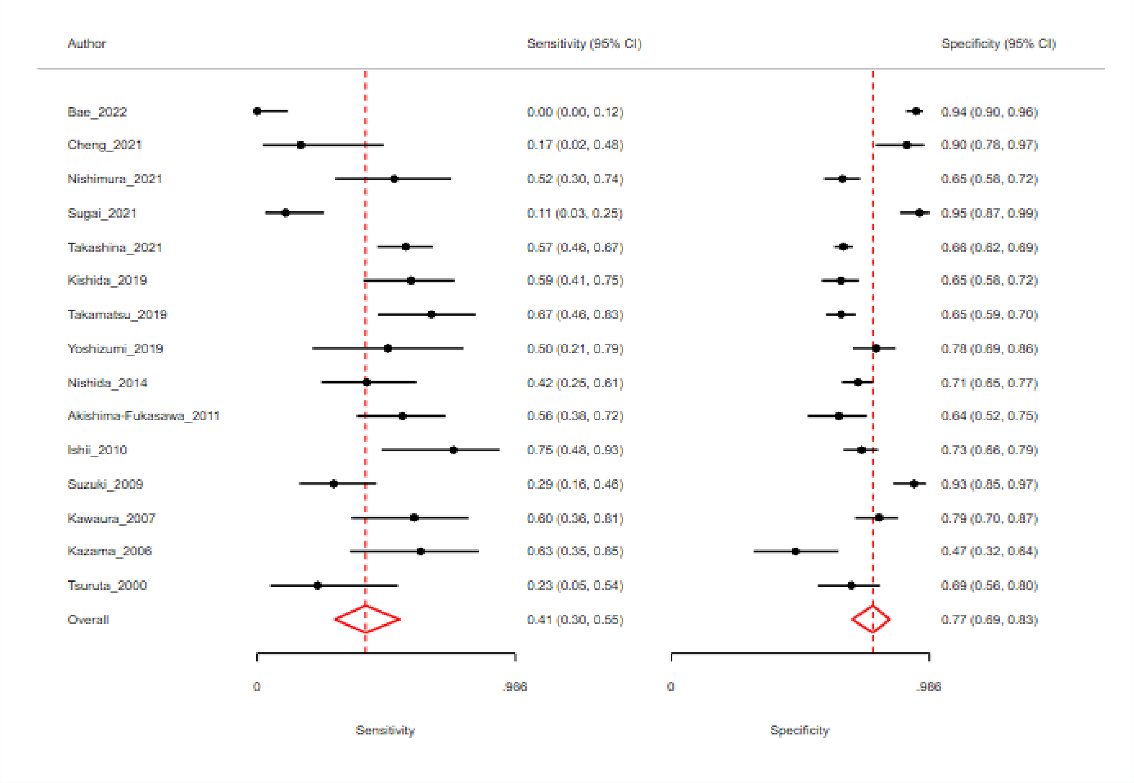

(B2) Elastic staining for VI in limited cases

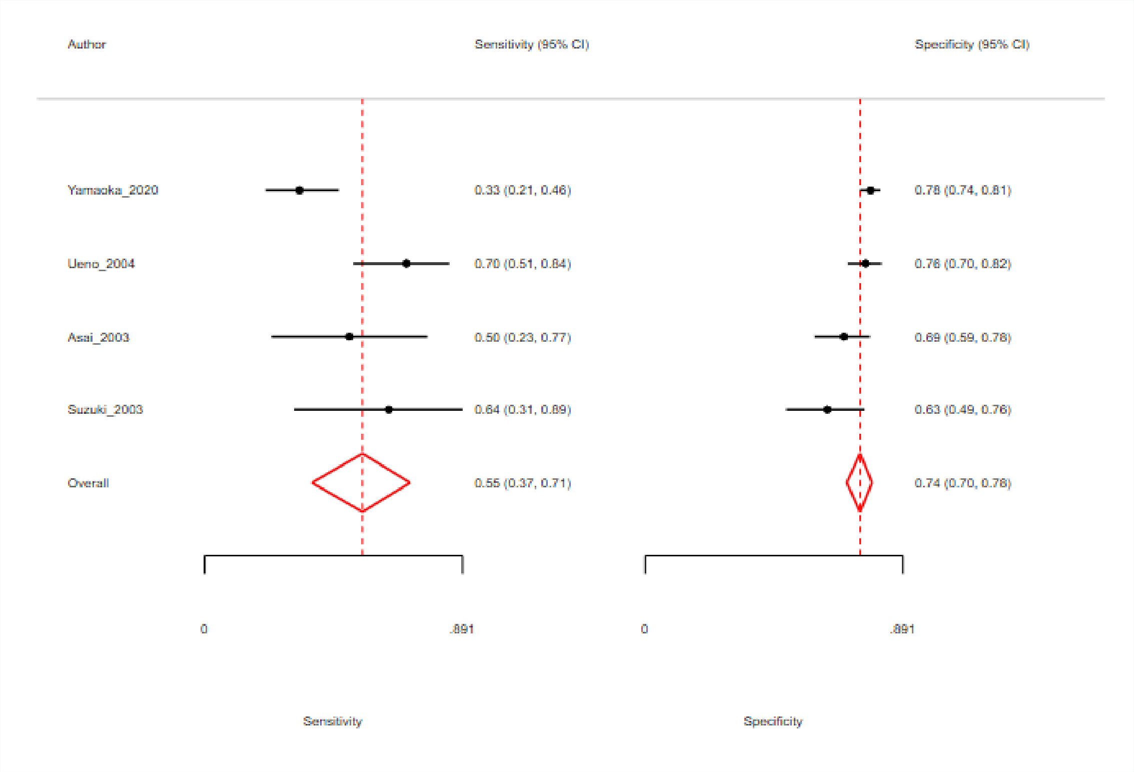

(C1) Additional staining for LVI in all cases

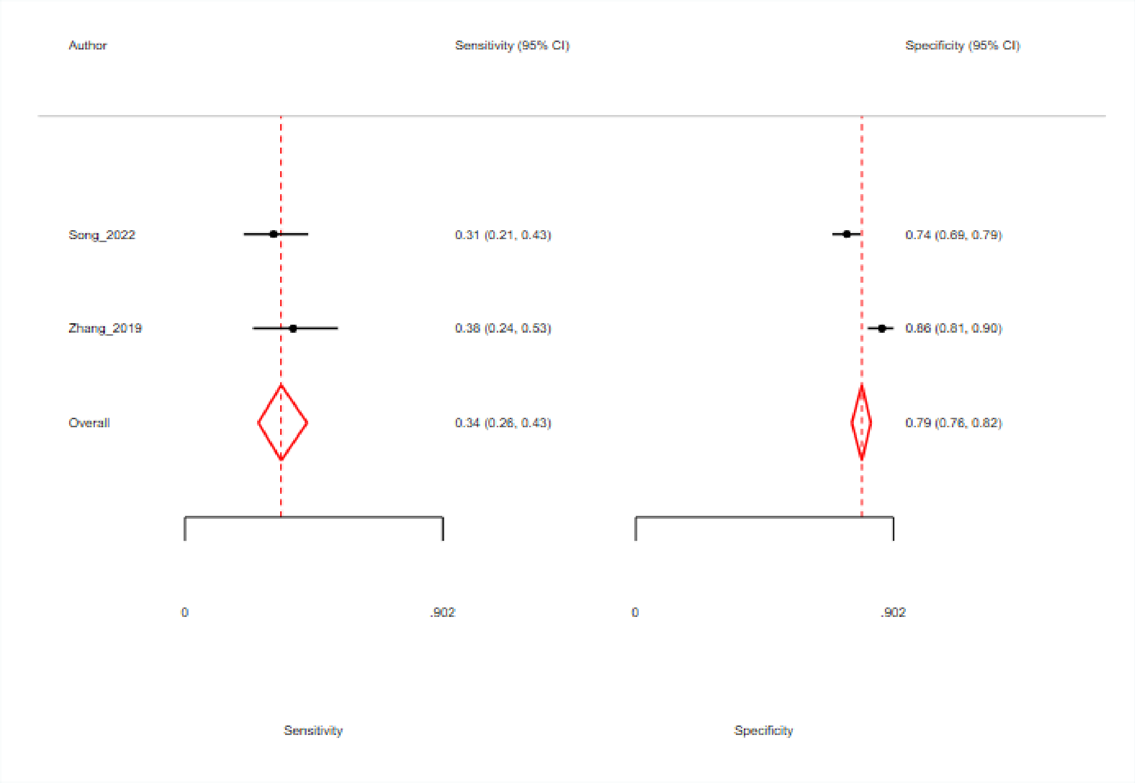

(C2) Additional staining for LVI in limited cases

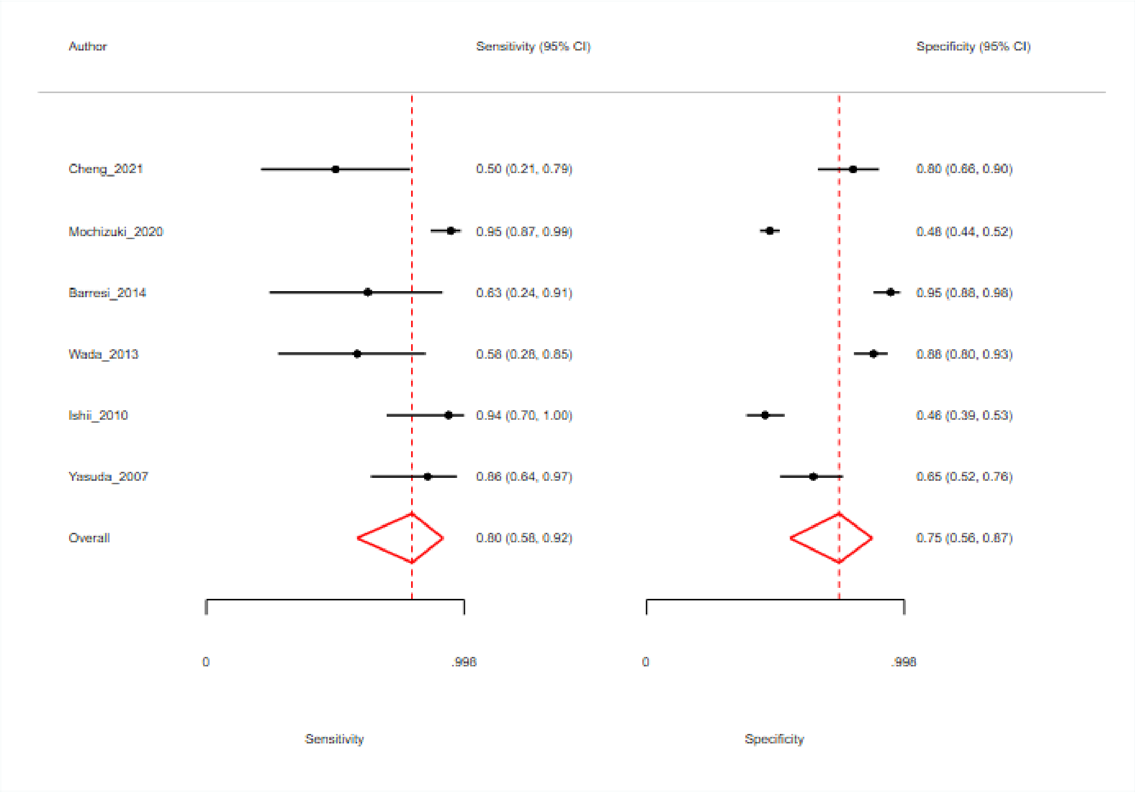

Supplementary Table S2. Sensitivity analysis

Sensitivity analysis	Invasion	Stain	No. of studies	Sensitivity	95% CI	Specificity	95% CI
Only our criteria	LI	HE	22	0.52	0.39–0.65	0.85	0.77–0.90
		D2-40	15	0.58	0.44–0.70	0.81	0.73–0.87
	VI	HE	15	0.26	0.15–0.41	0.90	0.81–0.95
		Elastic	17	0.44	0.35–0.53	0.75	0.69–0.81
	LVI	HE	12	0.47	0.33–0.61	0.86	0.75–0.93
		Additional	6	0.58	0.33–0.80	0.81	0.68–0.90
Only surgical cases	LI	HE	22	0.51	0.40–0.63	0.82	0.74–0.89
		D2-40	13	0.59	0.45–0.72	0.81	0.71–0.87
	VI	HE	16	0.25	0.15–0.40	0.89	0.81–0.94
		Elastic	18	0.43	0.32–0.55	0.77	0.70–0.82
	LVI	HE	13	0.45	0.32–0.58	0.88	0.78–0.94
		Additional	7	0.71	0.44–0.89	0.75	0.59–0.86
Only primary surgery cases	LI	HE	5	0.66	0.37–0.87	0.72	0.47–0.88
		D2-40	3	0.44	0.20–0.72	0.92	0.81–0.96
	VI	HE	4	0.25	0.04–0.73	0.89	0.66–0.97
		Elastic	4	0.47	0.32–0.61	0.76	0.56–0.89
	LVI	HE	3	0.16	0.12–0.20	0.95	0.93–0.97
		Additional	1	0.63	–	0.95	–
The same patients	LI	HE	3	0.38	0.21–0.59	0.87	0.64–0.96
		D2-40	3	0.68	0.52–0.81	0.75	0.60–0.86
	VI	HE	2	0.43	0.32–0.55	0.71	0.67–0.74
		Elastic	2	0.22	0.12–0.35	0.81	0.77–0.85
	LVI	HE	3	0.49	0.23–0.75	0.90	0.58–0.98
		Additional	3	0.75	0.43–0.92	0.82	0.52–0.95
CD31	VI	EVG (alone)	9	0.35	0.26–0.46	0.79	0.72–0.86
		VB (alone)	7	0.54	0.41–0.66	0.71	0.61–0.80
		CD31 (including other additional staining)	6	0.13	0.02–0.54	0.95	0.81–0.99
		CD31 (alone)	2	0.39	0.22–0.53	0.93	0.91–0.95

CI confidence interval; HE, hematoxylin-eosin; LI, lymphatic invasion; LVI, lymphatic and/or vascular invasion; VI, vascular invasion
